# Looking for Rhizobacterial Ecological Indicators in Agricultural Soils Using 16S rRNA metagenomic Amplicon Data

**DOI:** 10.1371/journal.pone.0165204

**Published:** 2016-10-25

**Authors:** José R. Valverde, Sonia Gullón, Rafael Pérez Mellado

**Affiliations:** Centro Nacional de Biotecnología, CSIC. c/Darwin, Madrid, Spain; Free University of Bozen/Bolzano, ITALY

## Abstract

**Introduction:**

Biological communities present in soil are essential to sustainable and productive agricultural practices; however, an accurate determination of the ecological status of agricultural soils remains to date an elusive task. An ideal indicator should be pervasive, play a relevant role in the ecosystem, show a rapid and proportional answer to external perturbations and be easily and economically measurable. Rhizobacteria play a major role in determining soil properties, becoming an attractive candidate for the detection of ecological indicators. The application of massive sequencing technologies to metagenomic analysis is providing an increasingly more precise view of the structure and composition of soil communities. In this work, we analyse soil rhizobacterial composition under various stress levels to search for potential ecological indicators.

**General Biodiversity Indicators:**

Our results suggest that the Shannon index requires observation of a relatively large number of individuals to be representative of the true population diversity, and that the Simpson index may underestimate rare *taxa* in rhizobacterial environments.

**Taxonomical Classification Methods:**

Detection of indicator *taxa* requires comparison of taxonomical classification of sequences. We have compared RDP classifier, RTAX and similarity-based taxonomical classification and selected the latter for taxonomical assignment because it provides larger detail.

**Taxonomy-Based Ecological Indicators:**

The study of significant variations in common, clearly identified, *taxa*, using paired datasets allows minimization of non-treatment effects and avoidance of false positives. We have identified *taxa* associated to specific perturbations as well as *taxa* generally affected in treated soils. Changes in these *taxa*, or combinations of them, may be used as ecological indicators of soil health. The overall number and magnitude of changes detected in taxonomic groups does also increase with stress. These changes constitute an alternative indicator to measuring specific *taxa*, although their determination requires large sample sizes, better obtained by massive sequencing.

**Summary:**

The main ecological indicators available are the Shannon index, OTU counts and estimators, overall detection of the number and proportion of changes, and changes of specific indicator *taxa*. Massive sequencing remains the most accurate tool to measure rhizobacterial ecological indicators. When massive sequencing is not an option, various cultivable taxonomic groups, such as specific groups in the *Actinobacteria* tree, are attractive as potential indicators of large disruptions to the rhizobiome.

## Introduction

Soil is the substrate that sustains terrestrial life on Earth, constituting an essential resource for the maintenance of most life processes, not only via agricultural production, but also by means of organic matter decomposition and nutrient cycling. Soil composition includes minerals, organic matter and a rich symbiotic community of micro- and meso-organisms that play a crucial role in the life-sustaining ability of soil. Viewing soil as a living ecosystem allows definition of soil health (also referred to as soil quality) in terms of its capacity to sustain plant and animal productivity under changing conditions.

The diversity and evenness of the plant growth promoting bacteria (PGPB) and rhizobacteria (PGPR) communities has been reported to increase agricultural yield via a variety of mechanisms. Nitrogen fixing bacteria include symbiotic species and non-symbiotic species such as *Proteobacteria*, and *Actinobacteria*. Phosphate solubilizing microorganisms provide a biological rescue system for inorganic P and include bacteria and fungi. The most efficient belong to the genera *Bacillus* and *Pseudomonas* among bacteria, and *Aspergillus* and *Penicillium* among fungi. Other bacteria improve mineral and water uptake (NO_3_^-^, PO_4_^3-^ and K^+^) [1,2].

In addition to providing ready access to nutrients, rhizobacteria produce a broad array of antibiosis products and functions, some of which target plant pathogens and therefore protect the crop. On the other hand, some soil micro-organisms are naturally resistant to a broad range of antibiotics (the "antibiotic resistome"). Depending on this balance, some soils have long been known to restrict the activity of plant pathogens and are known as "suppressive soils". The onset of suppressiveness in a soil is usually associated with a disease outbreak triggering activation of disease-suppressor bacteria. *Proteobacteria*, *Firmicutes* and *Actinobacteria* have been consistently associated with disease suppression, with *Actinobacteria* being the most dynamic *taxa* when pathogenic fungi were added [1,3,4].

Soil health, depends largely on the composition and diversity of the rhizobacterial community [5,6]. The rhizobacterial community symbiotically adapts to specific substances secreted by the crop being cultivated. Bacterial diversity and soil health can be adversely affected by stresses throughout the cultivation periods [5], increased salinity [7], acidity [8,9], soil composition and climate changes [10,11], tillage [12,13], cultivation methods [14], pesticides and heavy metals[15].Proper soil management strategies can reduce negative effects and restore the balance in the soil bacterial ecosystem, increasing soil health. Organic farming may overcome or reduce these effects [5], although it may reduce yield as much as 20% [14]. Balanced fertilization has also been found to increase soil biomass and activity [16]. The physical properties of soil also affect the bacterial community. Bacteria depend on microhydration for cell transport and nutrient diffusion. Sandy soils increase the number of isolated micro-habitats without crossed competition [17].

To guide management decisions, soil health is assessed using measurable quantities known as environmental indicators. Among these, ecological indicators reflect the balance of biological processes in the ecosystem. As such, soil ecological indicators are essential to assess soil health, suppressiveness potential, contamination and potentially deleterious effects [18]. In this study, we explore potential ecological indicators related to the rhizobacterial community. Such indicators should directly reflect the role of major biological determinants of soil health. Additionally, an ideal ecological indicator should be pervasive, display a rapid and proportional answer to external perturbations and be easily and economically measurable.

In the absence of extensive rhizobacterial composition data, several indexes have been used as overall indicators of soil diversity and richness. Frequently used indicators are the Shannon and Simpson indexes, operational taxonomic unit (OTU) counts and richness estimators, such as ACE or CHAO1. Both the Shannon and Simpson diversity indexes reflect the uniformity of species abundance. OTU counts provide a direct estimation of genetic richness, but they require very large sample sizes. Partial OTU counts suffer from a strong dependence on sample size and may be compared only with similar sample sizes; they are usually reported together with their corresponding rarefaction curves. Richness estimators (CHAO1 and ACE) predict diversity from partial measures of OTU counts. They also depend on sample size, although their values can stabilize earlier, reducing sample size requirements [19].

Next generation sequencing (NGS) facilitates the analysis of rhizobacterial populations in greater detail, up to, in a few cases, practically saturating the existing diversity in a given soil [20]. NGS studies have shown that soil diversity is far greater than originally expected, in the order of several thousand species [14,15]. Sequences can be assigned to known *taxa* using a variety of methods, although typically, a large fraction (~30%) of the sequences collected cannot be ascribed to any known *taxa*.

The availability of this new NGS data facilitates the identification of novel and more specific ecological indicators.

In this study, we have considered the minimum sample size required by general complexity and diversity indicators, such as the Shannon and Simpson indexes, to produce realistically representative estimates.

We have also considered the existence of taxonomic groups that may act as ecological indicators. We have compared rhizobacterial communities subject to different perturbations to identify associated taxonomic ecological indicators. Aiming for maximal generality, we paid special attention to groups that are commonly present in soil and that respond consistently to external influences. If such a group can be found, then small samples or relatively simple, specific tests might be designed to assert soil health.

Finally, any indicator should be economic and easy to determine, so that corrective actions may be taken swiftly. Since NGS technology is not pervasive yet, we also paid attention to cultivable rhizobacteria, as they may be used in resource-starved environments.

## Materials and Methods

### Sources of data

In this study we make use of data gathered in Lérida (Spain), Toledo (Yunquera and Calera y Chozas, Spain), Guadalajara (San Fernando de Henares, Spain), Zaragoza (Ejea de los Caballeros, Spain), [15,21,22,23] and Germany [24] (accession numbers PRJEB4333, SRP001013, SRX063207, SRA009281, SRA022075). These studies encompass a variety of cultivations (cotton, maize, Bt Maize, grasslands) in different locations and soils, during one or more cultivation cycles (from one up to four consecutive years), and climate conditions. In all cases, the studies contrasted the effect of a variable number of external influences (treatment with various herbicides or herbicide combinations and management methods) with corresponding controls under similar conditions. Under these experimental set ups, variations observed within the same experiment are due to differences in treatment, while differences between experiments are due to the environment.

### Metagenomic analysis

In all cases, soil samples were taken to analyse the rhizobacterial composition using metagenomics and 454-based pyrosequencing. The DNA was extracted and tagged with multiplexing identification tags, a hyper-variable region of the 16S rRNA (V6 or V3-V5) was amplified and pyrosequenced as described previously. Sequences obtained were quality and noise-filtered as described in the corresponding papers [15, 21–24].

Taxonomical classifications were obtained comparing every read against the reference Silva and RDP databases using Blast followed by taxonomic assignment using MEGAN as previously described [15]. Additional taxonomical classifications were computed using RDP Classifier with the stock training set (as implemented in QIIME[25]) and using RTAX [26] with the VAMPS database corresponding to each experiment. OTU-based analysis was conducted using QIIME to group reads into OTUs at the 97% similarity level, and to perform an alpha-diversity analysis computing the observed diversity, ACE, Chao1, Shannon and Simpson diversity indexes.

### Dependence of Shannon and Simpson indexes on sample size

To test the dependence of the Shannon and Simpson indexes on sample size, rarefaction curves for each index were computed using QIIME. After preliminary analyses, we settled on analysing the dependence of the Shannon index using a maximum depth of 50,000 individual sequences and 200 steps with a step size of 250 sequences, and the dependence of the Simpson index using up to 3,600 sequences and 50 steps with a step size of 80 sequences.

The sample size values corresponding to 95% and 99% of the final value obtained for each index in each sample were recorded, and the asymptoticity of the observed values was estimated computing the relative distance between the final sample size and the size that recovered 95% and 99% of the final value. Experiments with a ratio > 0.7 show less than 5% or 1% change of the indicator value in the final 70% of the rarefaction curve. The number of sequences needed in these experiments to reach 95% and 99% of the final value represents the sample size at which each index reaches a value within 5% or 1% of the final value. The average, standard deviation and confidence interval (C.I.) of the mean were computed to delimit the sample sizes needed to achieve significance.

### Comparison of taxonomical classification methods

The complete datasets, consisting of samples collected under various environmental conditions, were classified taxonomically as described. The total number of reads classified and the number of different taxonomic groups identified at each taxonomic level by each method were compared to check for differences in classification power, resolution and taxonomical detail.

RDP Classifier and RTAX provide imputation for bacterial sequences. Similarity-based classification can additionally find similarities to non-bacterial groups, as well as to unclassified and unspecific taxonomic groups (according to NCBI's taxonomy). These non-specific and non-bacterial groups were removed prior to comparison with the other classification methods to avoid biases. Taxonomic nomenclature has been checked against the list of prokaryotic names with standing in nomenclature (LPSN) [27].

### Statistical analysis

The frequencies of each taxonomic group at each taxonomic level for each experimental set-up were compiled and converted to percentages for further study and comparison.

The average composition in each *taxa*, its corresponding standard deviation, and its normalized standard deviation (NSD or coefficient of variance, *NSD = σ /* $\bar{x}$), were computed to identify their relative conservation, their proportional presence, and their variability under the range of environmental conditions considered.

Canonical Correspondence Analysis (CCA) was used to look for trends between experimental fields and taxonomic groups at each level and for each classification method. Next, the fields were grouped according to treatment. The resulting groups were used to search for indicator species using the Indicator Value method proposed by Dufrêne and Legendre [28] as modified by De Cáceres [29].

More specific tests were carried out comparing samples subjected to major external disruptions exclusively to their corresponding control. We selected samples that were larger, used the same 16S rRNA hypervariable region and that corresponded to agricultural fields treated with pesticides. Some pesticides have been reported to have an early impact on the rhizobacterial community, a long lasting effect, or both. Perturbations having an early effect on the rhizobacterial community were studied on the samples collected early after application (t1). Perturbations with a long-lasting effect were analysed on late-collection samples (t2). The treatments chosen correspond to acetochlor plus terbuthylazine with (ATG) or without (AT) glyphosate, pethoxamide and glyphosate (PG), mesotrione plus s-metolachlor and glyphosate (MSG), aclonifen plus isoxaflutole and mesotrione (AIM) and glyphosate (G). The early and late effects of each treatment has been described previously [11, 15, 21–24]

The relative proportions of *taxa* common between treated soils and their respective controls were compared using the likelihood based G-test followed by subsequent *post-hoc* tests (Fisher's exact test and χ^2^ test) applying the Holm-Bonferroni correction for multiple comparison to identify *taxa* whose proportion had changed with P < 0.05.

## Results

### Dependence of Shannon and Simpson indexes on sample size

**Fig 1:**
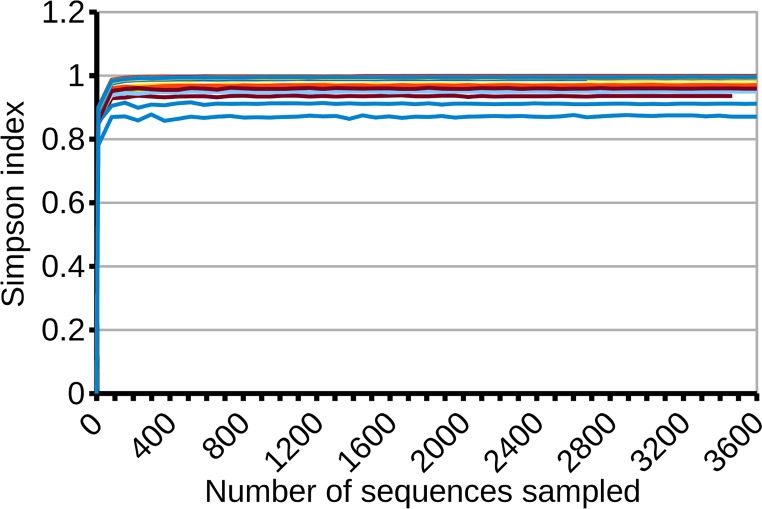
Stabilization of the rarefaction curves for the Simpson index. The rarefaction curves use a step size of 80 individuals and have been plotted only up to 3,600 individuals to facilitate the visualization. Each of the 82 samples is plotted in a different color. Stabilization is achieved with very small sample sizes, indicating that Simpson's index responds mainly to major groups and is largely independent of rare *taxa*.

**Fig 2:**
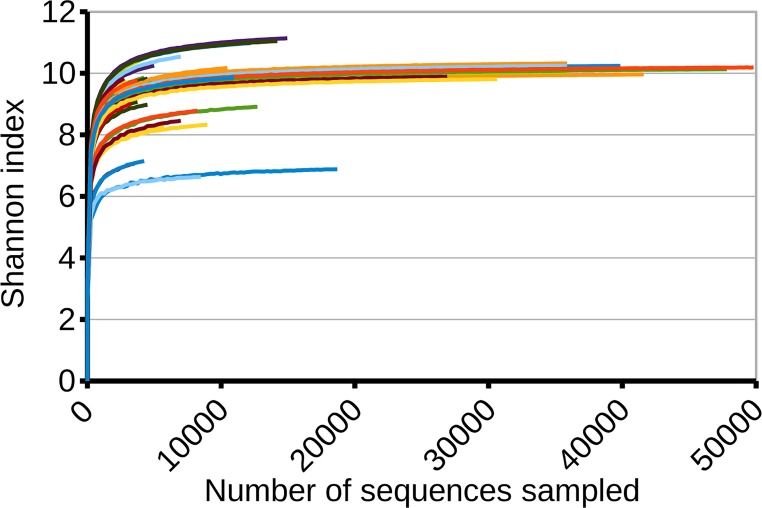
Stabilization of the rarefaction curves for the Shannon index. The rarefaction curves use a step size of 250 individuals and have been plotted only up to 50,000 individuals to facilitate visualization. Each of the 82 samples is plotted in a different color. Stabilization is achieved with large sample sizes, indicating that Shannon's index may be more responsive to rare *taxa* than Simpson's but at a higher cost in individual reads.

The dependence of the Shannon and Simpson indexes on sample size is plotted as rarefaction curves in Figs 1 and 2. The Simpson index tends to its asymptotic value with very small sample sizes: using a step size of 80 sequences, on average 153±0.1 (mean ± 95% C.I.) sequences were required to recover 99% of the final, asymptotically-stabilized value. This makes the Simpson index a useful tool when considering very small numbers of individuals/sequences, but also implies that it will not change with sample sizes larger than a few hundred samples, thus ignoring any additional information.

The Shannon index, required a larger number of individual reads to approximate its asymptotic value. Using a step size of 250 sequences, when all 81 samples were considered, the average numbers of reads needed were 2,290±11 and 5,829 ±46 to recover 95% and 99% of the final value. We can consider only the samples that have reached or are very close to asymptoticity by selecting those that have a relative change of less than 5% in the last 50% of the rarefaction curve. There are 41 samples that meet this criterion, and when these are considered, the number of sequences needed to recover 95% and 99% of the final value, become 3,374±16 and 9,654±73. We can increase the asymptoticity requirements to select only the samples with a change of less than 5% of the index value in the last 70% of the rarefaction curve. In this case, we end up with only 14 samples, where 95% and 99% of the final value can be recovered with 5,239±14 and 1,8542±97 individual observations. These findings suggest that a relatively large number of individuals are needed to obtain accurate estimates for the Shannon index and that, below these sample sizes, Shannon index values may be comparable only for very similar sample sizes.

### Analysis of taxonomic assignment methods

We have performed a taxonomic analysis to characterize potential taxonomic groups that might serve as ecological indicators. To perform this analysis we first determined the method best suited for our purposes by comparing the main methods proposed to identify taxonomic groups in metagenomic samples.

Taxonomic classification was performed using three methods: similarity-based (Blast + Megan), knowledge-based (RDP-classifier) and consensus-based (RTAX). Results are summarized in Table 1.

**Table 1:** Number of different *taxa* identified at each taxonomic level by each method.

	Consensus-based		Knowledge-based		Similarity-based	
	All	Bacteria	All	Bacteria	All	Bacteria
**Kingdom**	2	1	1	1	3	1
**Phylum**	36	34	35	35	73	59
**Class**	47	43	87	87	93	69
**Order**	98	98	187	187	162	155
**Family**	232	232			298	265
**Genus**					745	713
**Species**					1724	1697

The total number of different *taxa* identified by each method considering all samples together is reported. Knowledge-based classification was obtained using the RDP classifier and identified only bacterial sequences. Consensus-based classification used RTAX and a reference database to classify sequences employing information content to obtain a consensus reference assignment to identify bacterial and organelle sequences. Similarity-based classification used BLAST against a reference database followed by taxonomy assignment with MEGAN.

Only Similarity-based classification identified additional non-bacterial reads present in the samples.

The knowledge-based method has been trained to identify only bacterial sequences down to the order level, thus being best suited for coarse-grained taxonomical studies. With the datasets employed (which encompass a wide range of sample sizes and environments), knowledge-based classification assigns a taxonomy to the lowest number of sequences from the three approaches considered. On the other hand, this method tends to identify more different bacterial groups at each of the levels considered.

The consensus-based method uses a reference database to compute a set of consensus references for assignment. This approach results in the identification of a lower number of unique bacterial groups. On the other hand, it classifies a larger number of sequences than the knowledge-based method (approximately 2.1 times as many), reaches the family level and, in addition, identifies sequences from organelles (chloroplast and mitochondria).

Similarity-based classification uses more extensive databases comparing each sequence against every reference and, as a consequence, identifies a greater number of taxonomic groups (including candidate bacterial divisions and non-bacterial groups), levels and sub-levels than the two other methods. The similarity-based method also classifies a larger number of bacterial sequences than knowledge-based methods (approximately 1.8 times as many) although not as many as consensus-based methods at the same levels. Similarity-based classification was selected for subsequent analyses as the best compromise because it was able to produce the greatest resolution and to identify a large proportion of sequences.

### Identification of common taxonomic groups

Changes of an ecological indicator may be small or large, up to the total absence of a relevant group. It must be noted however that, since the soil rhizobiome is expected to contain thousands of species, most rare groups may escape detection unless a very large number of reads is used.

**Fig 3:**
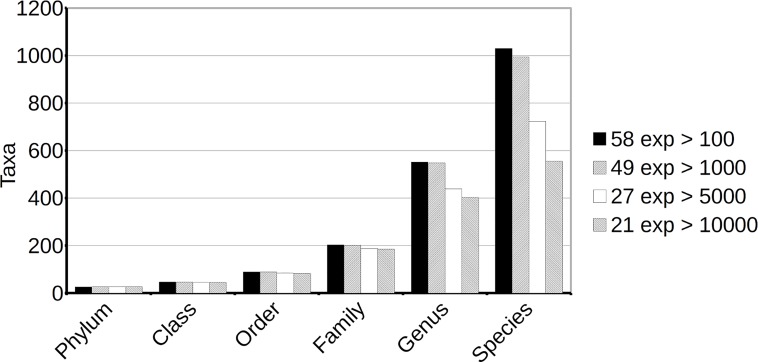
Total number of clearly identified taxonomic groups detected at each taxonomic level. Only *taxa* not to unspecific groups such as “environmental samples”, “unclassified”, “No hits” or “Not assigned”, nor to *Archaea* or *Eukaryota* have been considered. The figure shows that inclusion of additional information from experiments with smaller numbers of reads results in the identification of more taxonomic groups at every level. New *taxa* not present in larger experimental collections are likely rare *taxa*, and since they are detected with few reads, this suggests that there is a large proportion of rare taxonomic groups in agricultural soil.

**Fig 4:**
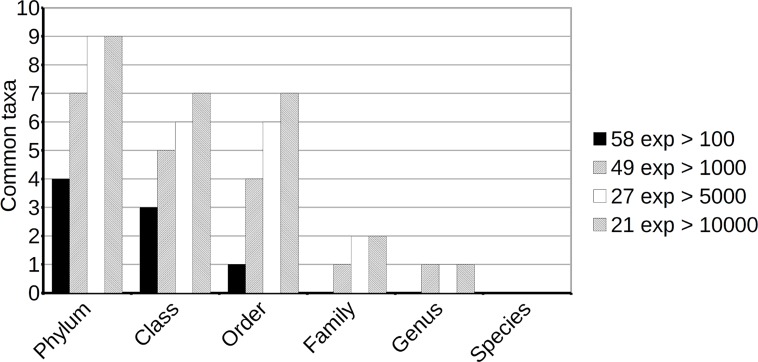
Number of clearly identified taxonomic groups common to all the experiments considered. Only *taxa* not assigned to unspecific groups such as “environmental samples”, “unclassified”, “No hits” or “Not assigned”, nor to *Archaea* or *Eukaryota* have been considered. The figure shows that inclusion of experiments with a smaller number of reads makes it more difficult to spot common groups (due to more rare *taxa* being missed) at every level. The reduction of common groups identifiable at lower levels suggests that a sample size of 10000 reads is not enough to capture all the biodiversity of soil samples and further supports the abundance of rare *taxa*.

We studied the *taxa* that had been observed and, which, among them, were commonly detected in experimental agricultural fields at each taxonomic level. Inspection of the results showed that similarity-based classification included many unspecific assignments. These generic groups may act as a “black box” that collates sequences from actually different groups at a given level. For these reasons, unspecific groups were not considered in the comparisons between experiments. The removal of these *taxa* decreased the number of groups identified by similarity assignment, but not enough to make any of the other classification methods preferable. The results are summarized in Figs 3 and 4.

We carried out analyses considering all experiments with more than 100 (58 soil samples), 1,000 (49 samples), 5,000 (27 samples) or 10,000 (21 samples) reads. As may be expected, increasing the number of soil samples considered (namely, including experiments with smaller sample sizes), and consequently the total number of reads included, also increased the total number of groups detected. This indicates that many groups correspond to rare *taxa*, and that they are detected or missed largely by chance (Fig 3). On the other hand, augmenting the size of the samples considered (and ignoring experiments with small sample sizes) results in an increase in the number of common groups detected. Smaller samples miss a larger number of rare taxonomic groups, which therefore cannot be identified as common to all samples. Consideration of only the larger samples facilitates the identification of additional common groups (Fig 4).

### Community composition

The extent of the perturbation that needs to be detected to discern external effects increases with the natural variability of a taxonomic group in the samples considered. The average presence of each taxonomic group showed a large variation, reflecting the unevenness of taxonomic distributions in natural soils. The results are provided as S1 Table. These results explore the average community composition and variability across the range of locations, times, cultivations and agricultural methods surveyed.

At the phylum level, the most frequent taxonomix groups identified, ignoring unspecific groups, were *Proteobacteria* ($\bar{x}$ = 0.34%/NSD = 0.32), *Actinobacteria* (26%/0.36), *Acidobacteria* (15%/0.44) and *Verrucomicrobia* (12%/0.57); at the class level they were *Actinobacteria* (33%/0.33), *γ-proteobacteria* (18%/0.51), *α-proteobacteria* (12%/0.73) and *Acidobacteriia* (12%/0.7); at the order level, they were *Actinomycetales* (28%/0.37), *Acidobacteriales* (15%/0.66), *Verrucomicrobiales* (7%/0.95) and *Rhizobiales* (5%/0.51); at the family level, *Acidobacteriaceae* (16%/0.69), *Nocardioidaceae* (8%/0.54), *Verrucomicrobiaceae* (5%/1.04) and *Pseudomonadaceae* (5%/0.73); at the *genus* level, *Acidobacterium* (10%/0.97), *Nocardioides* (6%/0.7), *Verrucomicrobium* (6%/1.08) and *Streptomyces* (6%/0.58); and at the species level, *E*. *coli* (9%/1.75), *P*. *aeruginosa* group (6%/0.84), *P*. *aeruginosa* (6%/0.84) and *L*. *gummosus* (4%/1.55). It must be noted that upper levels also include non-specifically imputed individual reads from lower levels.

### Identification of statistically significant changes

To investigate the potential association of specific taxonomic groups with external perturbations we performed a CCA of samples and species with respect to all the different treatments applied. The CCA was computed at each taxonomic level, and for each classification method. The results are provided in S1 Appendix. In each case, major components consistently showed that early vs. late sampling time and control or conventional management vs. pesticide use are major components segregating the samples.

The results of the analyses using the Indicator Value approach are included in S1 Appendix. Analyses with each of the three taxonomic classification methods identifies several potential indicator *taxa* at each level when all the *taxa* identified are included in the analysis. Many of the potential indicators correspond to unspecific *taxa*. When the analysis is restricted to consider only the *taxa* commonly present in the samples considered, no indicator is found at any level with any of the taxonomic classification methods.

To study which proportions of common *taxa* had changed significantly, we compared the taxonomic distributions of samples exposed to various pesticide treatments to their corresponding control at each taxonomic level. Only groups common to each control and test sample were considered. This restriction was applied to avoid false positives due to rare groups missed by chance. Significance was checked using the G-test, followed by *post-hoc* tests (Fisher's exact test and χ^2^ test) with the Holm-Bonferroni correction for multiple comparisons. The values obtained are listed at each taxonomic level and collection time (to allow distinction of early and long-lasting effects) in S2 Table and summarized below. At the phylum level most major groups were usually affected during the early response to external stress (*Actinobacteria*, Acidobacteria, Chlamydiae / *Verrucomicrobia*, *Firmicutes and Proteobacteria*, being the last two most commonly affected). When there were long-lasting effects, the groups most commonly affected were *Actinobacteria*, Acidobacteria, Chlamydiae / *Verrucomicrobia and Firmicutes*.

At the class level, the groups most commonly associated to early response were *Actinobacteria*, *Bacilli*, *α-*, *δ- and γ-proteobacteria* and *Verrucomicrobiae*, and with long-lasting effects, *Actinobacteria*, *Acidobacteriia*, *Bacilli*, *α- β- γ-* and *δ-proteobacteria* and *Verrucomicrobiae*.

At the order level, the groups most commonly associated to early response were *Actinomycetales*, *Bacillales*, *Sphingomonadales* and *Verrucomicrobiales*, and with long-lasting response, *Actinomycetales*, *Aeromonadales*, *Bacillales*, *Myxococcales*, *Enterobateriales*, *Pseudomonadales*, *Verrucomicrobiales* and *Vibrionales*.

At lower levels, the number of groups that are associated with the early and long-lasting effects of pesticides increase, but so does the presence of groups observed only in environmental samples without more detailed characterization (see S2 Table). At the extreme, at the species level, most of the variability is associated to *taxa* characterised only as proceeding from uncultured environmental samples. Among the well-known *taxa*, *E*. *coli*, the *E*.*cloacae* complex, the *P*.*aeruginosa* group and the *P*. *putida* group where the most commonly affected at the species level.

At all levels and times, the total number of *taxa* affected shows a large variability between treatments. These differences are consistent for each treatment across taxonomic levels, i.e. the treatments that show a larger number of affected *taxa* consistently do at all taxonomic levels. Global variation provides a direct measure of the general impact of the treatment on the rhizobacterial community.

## Discussion

Given the lack of a powerful direct ecological indicator, soil ecological studies have relied on indirect indicators of overall diversity and richness computed from limited experimental data. Experiments have used phospholipid fatty acid analysis (PLFA), terminal restriction fragment length polymorphisms (T-RFLP), single-strand conformation polymorphism (SSCP), denaturing/temperature gradient gel electrophoresis (DGGE/TGGE) and sequencing of a small number of 16S metagenomic sequences. The main soil health indicators currently in use are the soil chemical and physical properties, OTU content and the Shannon and Simpson indexes.

Soil physico-chemical properties will undergo little changes over time and may be considered as relatively invariant in most soils. Hence, while it may be used to compare different edaphic backgrounds, it is unlikely to change in response to external stimuli making a poor indicator of ecological changes. Actually, micro-habitat fragmentation in sandy soils results in greater richness without associated changes in the Shannon and Simpson indexes [17].

The sample-size dependence of direct diversity measures (OTU and OTU-predictors such as Chao1 or ACE) has already been studied. There is a large dependence of OTU counts on sample size, requiring large and uniform samples (on the order of 50,000 to 500,000 individual reads) to estimate the actual diversity of an agricultural soil. Diversity estimators, such as Chao1 or ACE, can reduce this dependence depending on the characteristics of the underlying population [19]. However, they still require relatively large sample sizes (usually in the order of at least 10,000 to 25,000 individual sequences) to reach asymptotic levels.

To further reduce the sampling size and facilitate experimental detection of external influences, the next obvious choice is to use coarser indicators. The Shannon and Simpson indexes are generally used to provide an estimation of richness and diversity in current studies. We have analysed the dependence of both indexes on the number of individuals examined.

Our results show that the Simpson index may provide a reasonable estimate of its actual population value with relatively small sample sizes (about 150 individual sequences) such as those collected in many non-NGS studies. However, recent NGS studies have shown that agricultural soils may host thousands of species. This suggests that Simpson's index, with its small sample size requirements, is actually responding only to major *taxa*, and may be less sensitive to changes in less-abundant *taxa*. Consequently, the Shannon index might reflect better the actual landscape of the soil rhizobacterial diversity.

The Shannon index requires at least an order of magnitude more information to be reliable. More than 5,000 individual sequences are required to obtain a value that might be within 95% of the actual population value (or more than 18,000 for 99%). According to our observations, when using smaller sample sizes, the Shannon index should be used cautiously and, preferably, to compare results only between experiments with similar sample size. Experiments with a much larger sample size may be comparable even with unequal sample sizes.

It should be noted that we have used a relatively coarse precision to build the rarefaction curves, and that the samples used do not represent the full spectrum of natural soils. The values reported for the mean should be considered only a rough approximation despite the small values of their confidence intervals. These C.I. values should be considered a measure of the relative dispersion observed, not as limits for the values of the average sample size.

Since the Simpson index stabilizes too early, the Shannon index seems more suitable to consider the rich information obtained from the analysis of modern experiments that yield larger sample sizes. On the other hand, once we have access to this rich information, it makes sense to search for other potential indicators. A case in point would be identification of an indicator taxonomic group. Such a group might permit elaboration of specific tests for the detection of major environmental changes with less effort and at a lower cost.

Previous work has shown the relative dependence of different bacterial *taxa* on environmental conditions. We have looked for taxonomic groups that may act as sensitive ecological indicators. A desirable group should be present in most, if not all, soils, behave consistently under external influences, should be easy to detect and, possibly, cultivable for cheaper detection in budget-constrained environments.

The analysis of various approaches to taxonomical classification confirms that similarity based assignment provides the most fine-grained results. This increased detail requires a higher computational cost. While RDP and RTAX only require a few seconds or minutes to classify a large dataset in a modern computer, running blast against the VAMPS, Silva or RDP databases may require an overnight, or longer, calculation. On the other hand, until more data is available, many individual sequences will remain unclassifiable introducing a bias in the analysis. In addition, most studies do not exhaust species diversity as this requires extensive sequencing (in the order of hundreds of thousands of reads). As NGS technology progresses and data accumulates, we may expect these shortcomings to be less relevant.

Despite current limitations, NGS remains the most accurate approach for metagenomic analysis. When using NGS data, similarity based taxonomy should be preferred whenever maximal detail is desired. However, RDP Classifier and RTAX provide a practical way to speed up analyses if practical concerns require a compromise for speed and if the sacrifice of the detail from the lowest taxonomic levels is acceptable.

The presence of unspecific groups used to annotate environmentally-collected metagenomic sequences further affects similarity based classification. The wealth of new data demands novel approaches to taxonomical classification. In this work we decided to use only well-characterized groups, clearly reducing the information considered. A better approach might be to classify novel reads into new, provisionally labelled, taxonomic groups to enhance taxonomic imputation of newly collected sequences. Such an approach would parallel OTU clustering followed by taxonomic imputation and suffers similar drawbacks: there is a strong dependence of similarity clustering on sample characteristics; 16S rRNA hypervariable regions may constitute a genetic continuum blurring cluster limits; the correlation of the genetic diversity of 16S rRNA sequences to overall genome diversity is unclear; and the relationship of percent similarity with the definition of taxonomic levels is still open to debate. Furthermore, OTU clustering followed by taxonomy assignment comports an initial step where several individual reads are grouped and all of them are arbritrarily assigned the taxonomy of the cluster centroid (the group's chosen representative). We have chosen to classify taxonomically each read independently to increase precision and avoid biases associated to OTU clustering.

Identification of common groups is hampered at lower taxonomic levels. This is most likely because the number of expected groups increases as we proceed towards the species level and, conversely, the number of individuals in any group will decrease. The proportion of extant, clearly defined taxonomic groups also decreases as we proceed towards the species level and, with current, reduced sample sizes, it is easy to miss and be unable to classify many lower level groups.

The CCA analysis of all the samples included in the analysis revealed two common trends irrespective of the taxonomic level and classification method used. Time is often associated to the first canonical coordinate, and control/conventional cultures tend to cluster separately on the second component. These results support the feasibility of identifying rhizobacterial ecological indicators of external stress.

The Indicator Value method has been successfully applied to test for indicator species using metagenomic data [30] of tilled vs. non-tilled crop production. Its application to our datasets is questionable as the classification of pesticide impact is normally based, among other parameters, on the effect on taxonomic diversity at higher (usually class or phylum) levels. Still, since CCA shows a clear separation of control and conventionally cultivated samples, it might be useful to pool these samples into a common group and look for indicators comparing it to the other samples separated by treatment.

The analysis identified many potential indicators, usually grouping samples in accordance to previous classifications. These indicators are, however, subject to another criticism: current metagenomic studies explore only a minor fraction of the existing species (as deduced from OTU and estimator values). It is impossible to rule out that any absent species have not been missed by chance. If a species has been missed by chance, it may be erroneously identified as a false positive indicator. When all non-common species were removed from the study, no indicators were found at any level. This does not invalidate the indicators found, but as all of them might potentially be a false positive, we cannot rely on them without additional confirmation.

The Indicator value approach groups many samples by treatment and relies on group size for the effects of non-treatment differences to cancel out. In this case, variation between experiments might plausibly obscure variation within independent experiments. We can overcome this by fixing all non-treatment variables. We have selected larger samples and compared each treated sample with its respective control from the same location, soil and, time/climate cycle.

An interpretation of comparative taxonomic data is difficult due to various reasons. The treatments considered have varying degrees of impact on the soil ecosystem, thereby challenging the interpretation of the changes observed. They also have different time dependences, with some treatments displaying an initial impact that can quickly be recovered, while others may display small initial effects that accumulate and increase with time. Lastly, the information available pertains to a reduced number of soils that do not reflect the overall variability potentially existing in agricultural soils.

With the taxonomic information currently available, the analyses show that there are many potential indicators associated to specific treatments. Many of these correspond to *taxa* catalogued generically as environmental samples, suggesting that many additional indicators are possibly present, although they have not been catalogued yet. Our results indicate that our current knowledge is unlikely to identify any rhizobacterial group at the various levels that is always associated to all aggressive conditions. Nevertheless, we have been able to identify at each level the groups most commonly associated with early or long-lasting aggression and these provide a useful starting point to diagnose deleterious effects in the soil environment.

Until more data is collected, it is sensible to use changes in the proportion of any of these groups, or their combinations, as indicators of alterations in soil health. NGS is nowadays the most accurate method to explore the soil bacterial community, but depending on resources, it may compensate to compromise for less accurate detection methods. In the most restricted environments, detection of cultivable groups at higher levels may be an optional approximation.

Choosing an appropriate group that may function as ecological indicator in the absence of NGS data is not easy. From the major *taxa* present in all soils, groups at various levels in the trees of *Verrucomicrobia* and *Actinobacteria* show a response to external influence, pervasiveness (within our samples) and low variability (as measured by NSD) so that their proportional variations in response to external influences should be easier to detect. Groups in the *Proteobacteria*, and more specifically, *δ-proteobacteria* tree are also commonly affected. At the species level, *E*. *coli*, *Enterobacter cloacae* and *Pseudomonas* are frequently affected in agreement with previously published work and, combined, provide a good indicator of long-lasting damage to the soil ecosystem.

There are easy and efficient methods to determine the presence of *Actinobacteria* in soil [31], making them attractive as potential indicators. In addition, the proportion of *Actinobacteria* in any given soil tends to stabilize under normal conditions [32] being resilient to pH changes (which strongly affects other major *phyla*) [9,13]. They are producers of antimicrobial compounds [6] and they are normally present in greater proportions in agricultural soil than *Verrucomicrobia*. Hence, the determination of *Actinobacteria* should be easier and require smaller samples.

We have observed early and late changes in *Actinobacteria*, showing an increasing effect with more aggressive treatments, in the rhizobacterial communities of maize and cotton fields treated with various herbicides or herbicide combinations [10,15,23,33]. Other authors have reported similar changes associated to tillage and crop rotation/succession [12]; larger abundance of *Actinobacteria* in suppressive soils with greater changes after the addition of fungal pathogens [4]; long term fertilization and intensive herbivory plus mowing in grasslands [34]; intensive coffee farming [14]; desert versus cultivated soil [35]; and different depths of permafrost soil samples [36].

This suggests that relative changes in the proportion of *Actinobacteria* groups (for instance, actynomycetes) may act as potential ecological indicators when NGS technology is not available.

However, given the relative scarcity of data available, these observations should be taken with caution until more information is collected. Our knowledge of the rhizobiome is still too limited and, as data accrues, new information will need to be considered, possibly demanding a reconsideration of these observations.

An alternative approach is to consider the total number of changes observed: generally speaking, more aggressive treatments affect a larger number of *taxa*. This suggests that the total number of significantly altered taxonomic groups and the magnitude of the change in their proportional presence may also be used to define the impact of external influences. A statistical test of the number of changes weighted by their relative magnitude should provide a useful measure of the impact of external influences on soil health. Additional metagenomic data is needed to parametrize weights and measure correlations. The main drawback is that, to be useful, many sequences are needed to properly sample the rare microbiome.

Comparison of complete rhizobiomes will yield more information and, with the extension of sequencing technologies, may become the option of choice. With the characterization of the complete rhizobiomes from sufficient diverse samples, the identification of a better *taxon* or group of *taxa* that may act as indicator will also become easier.

## Conclusion

We have considered several potential ecological indexes based on the current availability of metagenomic data. Our results show that the Shannon index requires large sample sizes (~5000 individuals) and the Simpson index likely underestimates rare species. OTU-based methods to measure diversity require even larger sample sizes, although they may be reduced by the use of conscientious selection of the CHAO1 or ACE estimators. Taxonomy-based indicators may provide a sensible alternative. This approach requires taxonomic imputation of individual sequences, which -according to our results- is best achieved using similarity-based methods, although other approaches such as RDP or RTAX may provide a coarser but faster short-cut for huge datasets. Choosing one or more groups to use as ecological indicator is limited by the relatively reduced NGS data available. The Indicator Value approach can provide useful information in controlled experiments or when enough data is available, and should become a routine procedure in future metagenomic analyses. When tit is not applicable, a more specific case-control approach such as the one described here should be more sensitive.

As a rule, NGS technology is currently the most accurate tool to explore rhizobacterial communities, allowing identification of ecological indicators as changes in specific *taxa* or in the overall number and proportions of changes. When NGS is not an option, various *taxa* in the *Actinobacteria*, *Verrucomicrobia* and *Proteobacteria* trees may be selected as indicators at various taxonomic levels. In particular, relative changes in the proportions of *Actinobacteria* are easy to detect and commonly associated to major external perturbations.

Most of the data currently available describes the rhizobacterial communities under normal agricultural practices and there is a scarcity of contrasted data on the effects of other major external perturbations. We have considered mainly soils treated with pesticides having a well-known, proven impact, and our results agree with other observations. However, all the results reported in this work should be revised as additional data collected from ecologically affected soils becomes available.

## Supporting Information

S1 Appendix Preliminary statistical analysis. The results of CCA and Indicator Value analyses obtained with each of the three classification methods are provided in separate subfolders as a compressed Zip archive.
(ZIP)

S1 Table Taxonomic groups observed. Details of all the taxonomic groups observed in the samples. The values reported are organized by taxonomic level and sample. Both, the frequencies (total number) of reads assigned to each taxonomic group and the percent that a group represents at each level in each sample are provided.
(XLS)

S2 Table Statistical analysis of taxonomic changes observed. The table contains the statistical analysis (G-test followed by *post-hoc* Fisher's exact and χ^2^ tests) of taxonomical changes observed between each sample subject to an external stress and its respective control. Changes have been classified by taxonomic level, experiment, stress applied and time of collection (early or late). Early samples are expected to reflect responses to rapid effects, and late samples are expected to reflect responses to long-lasting effects. Significant differences identified by the G-test are marked with three stars (***) for easier identification.
(XLS)
