# Supplementary figures and images for: Looking for Rhizobacterial Ecological Indicators in Agricultural Soils Using 16S rRNA metagenomic Amplicon Data
Source: PLoS One. 2016 Oct 25;11(10):e0165204. doi: 10.1371/journal.pone.0165204 (PMC5079562; doi:10.1371/journal.pone.0165204)

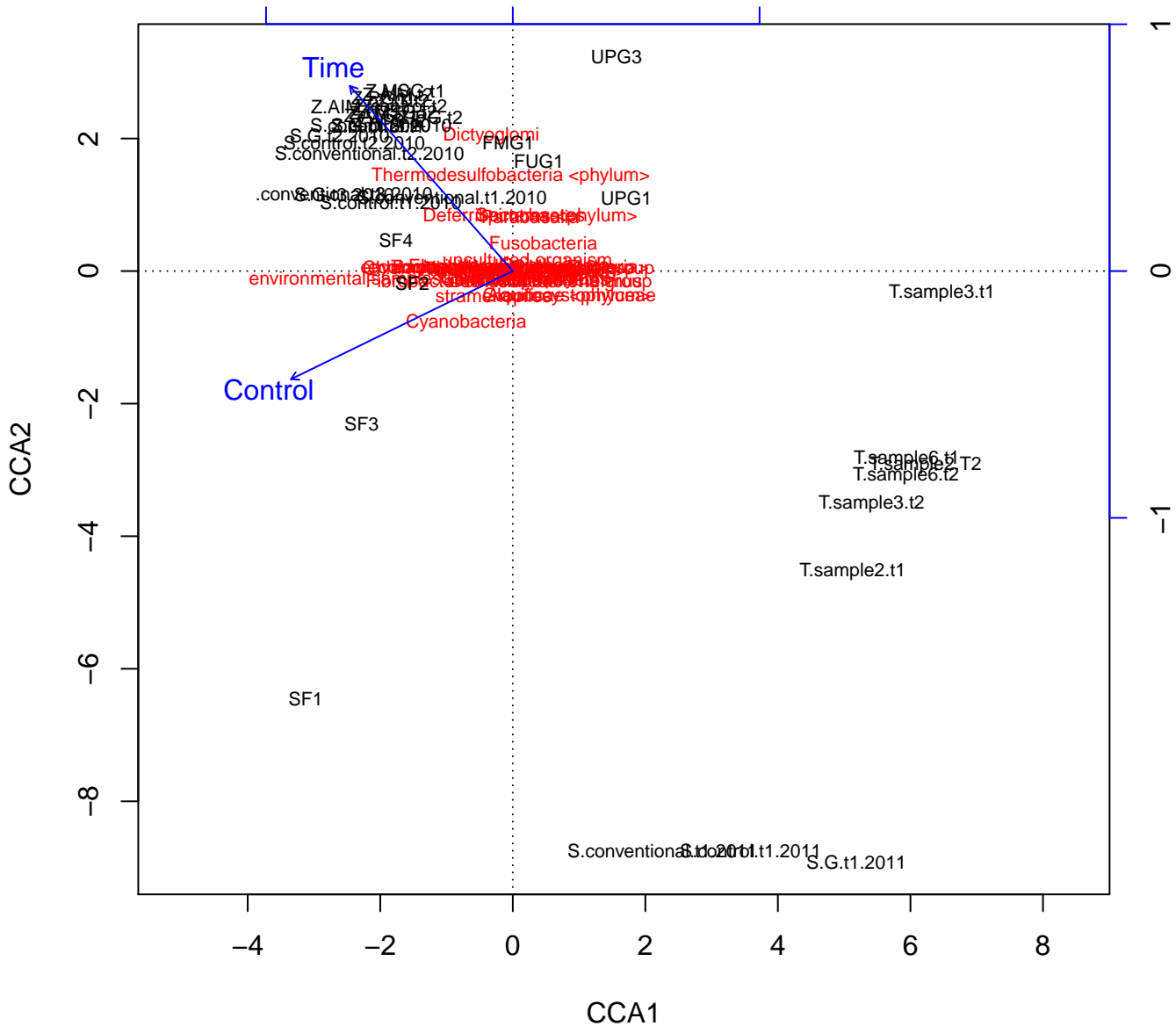

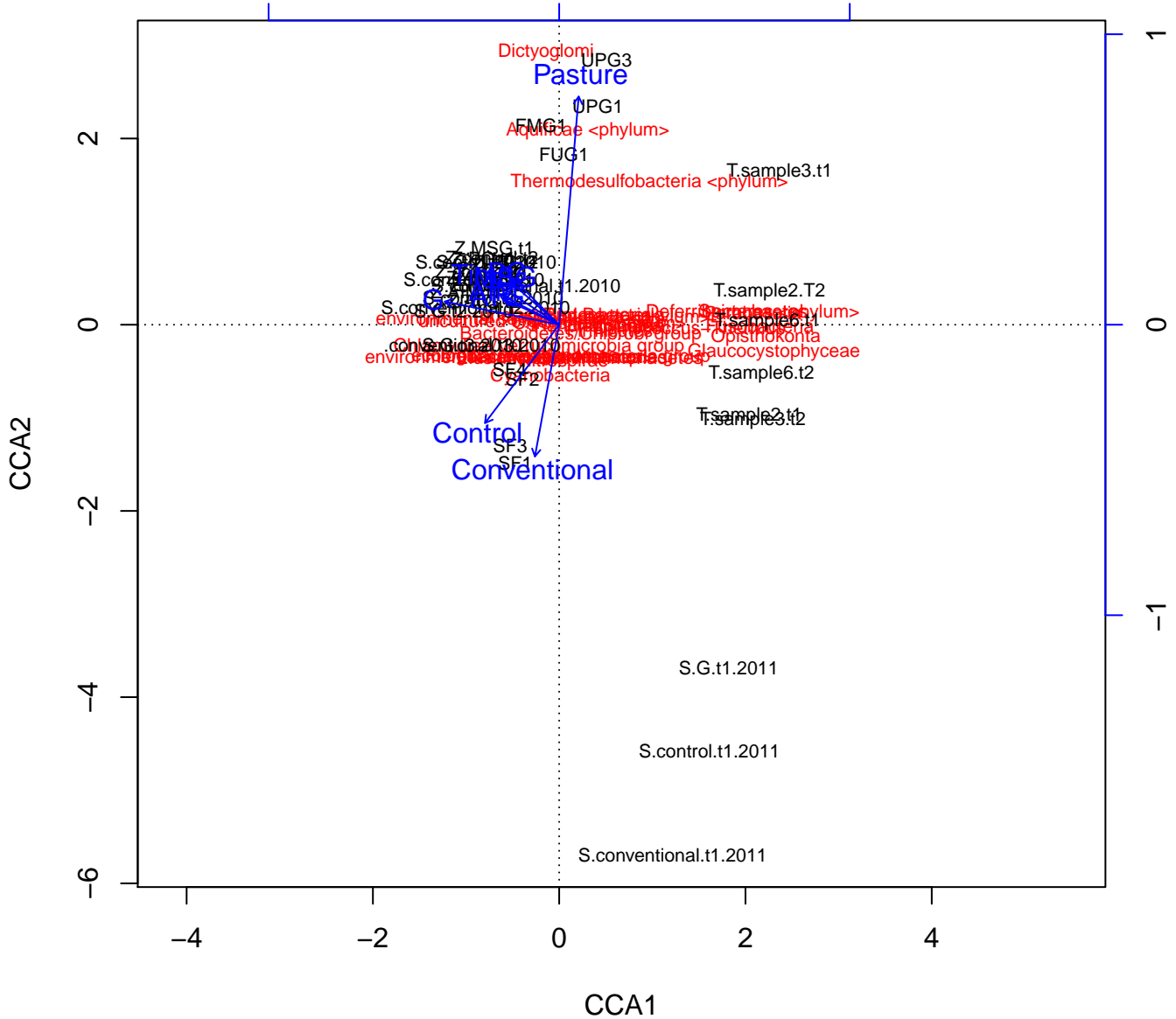

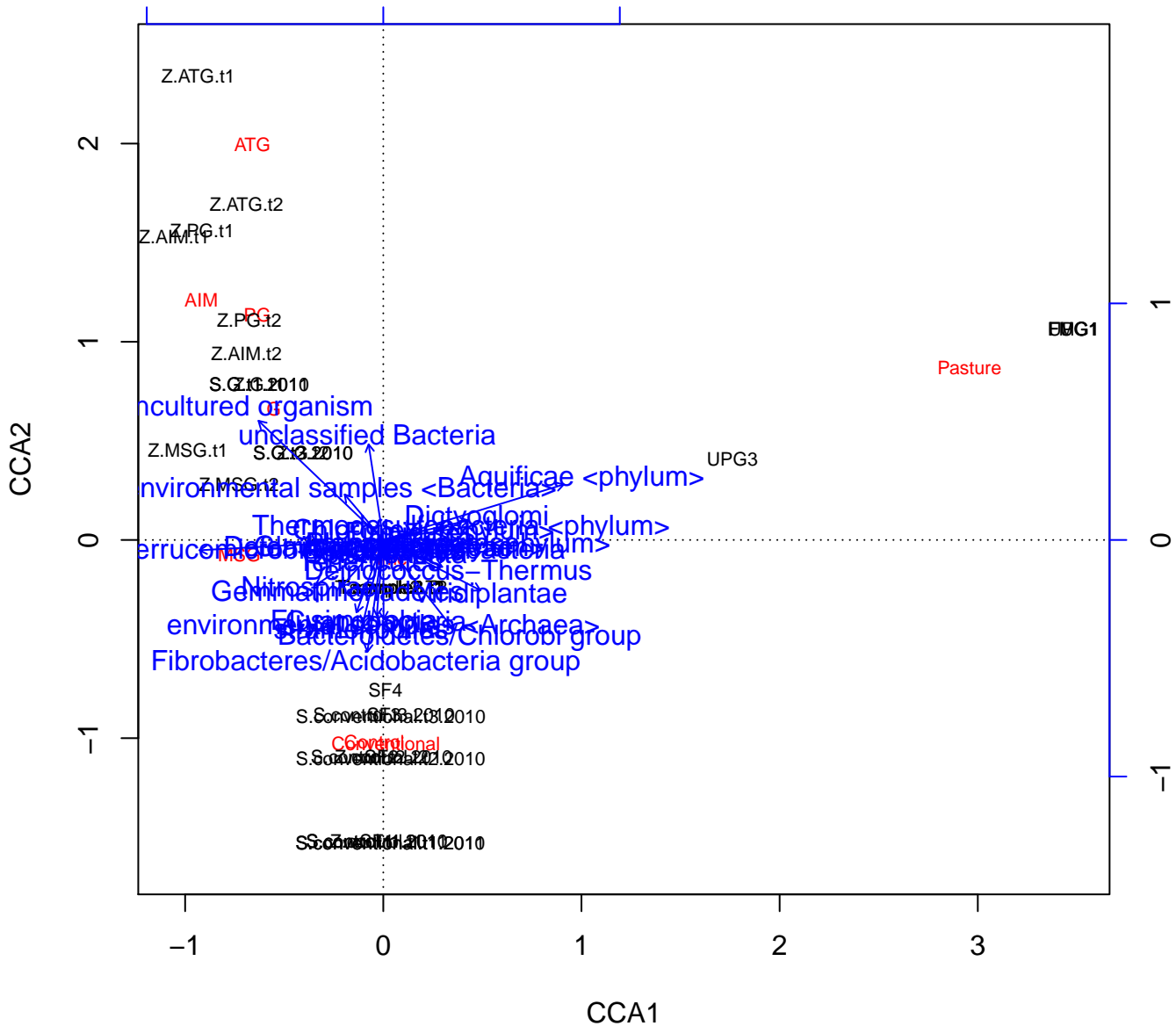

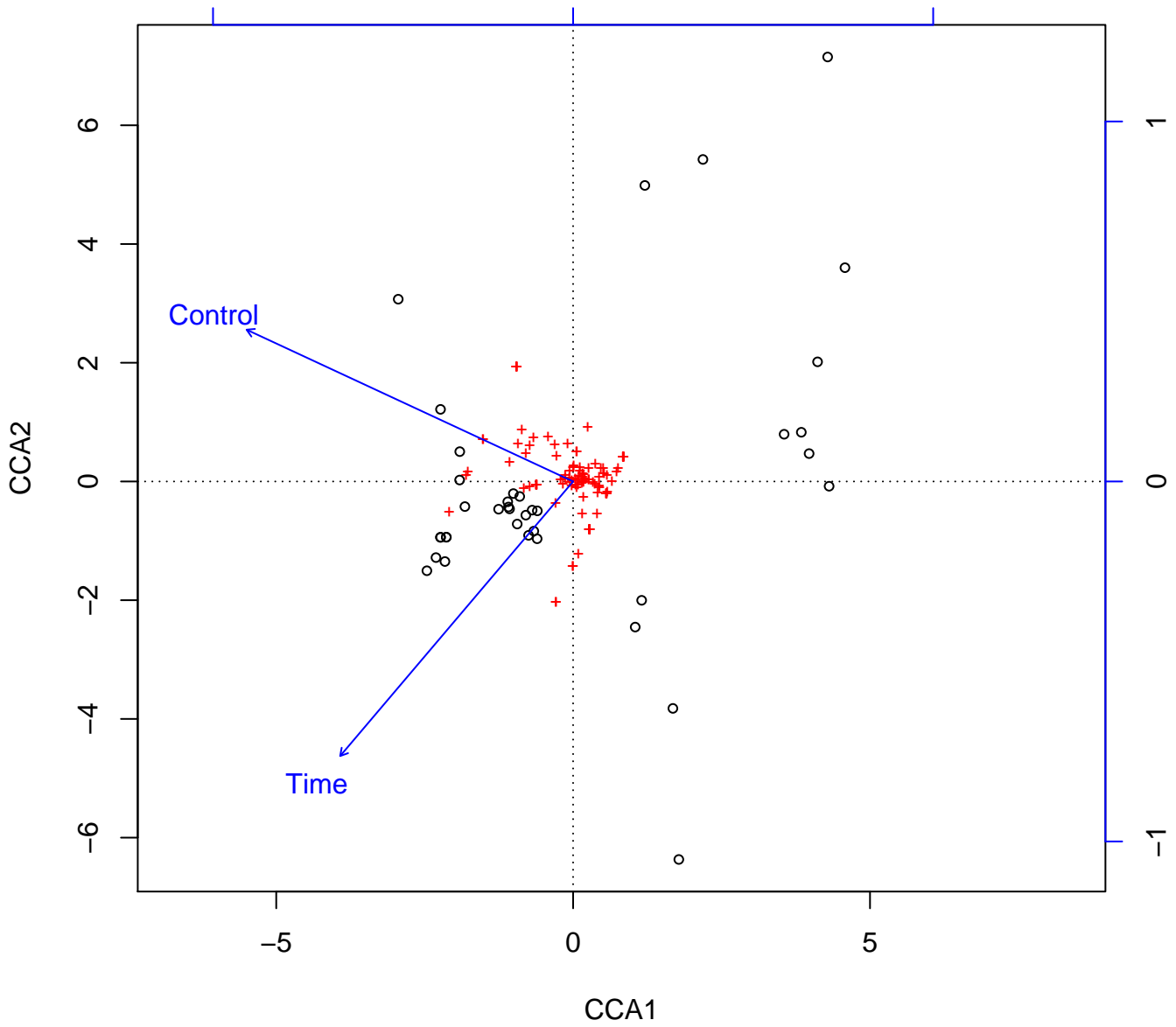

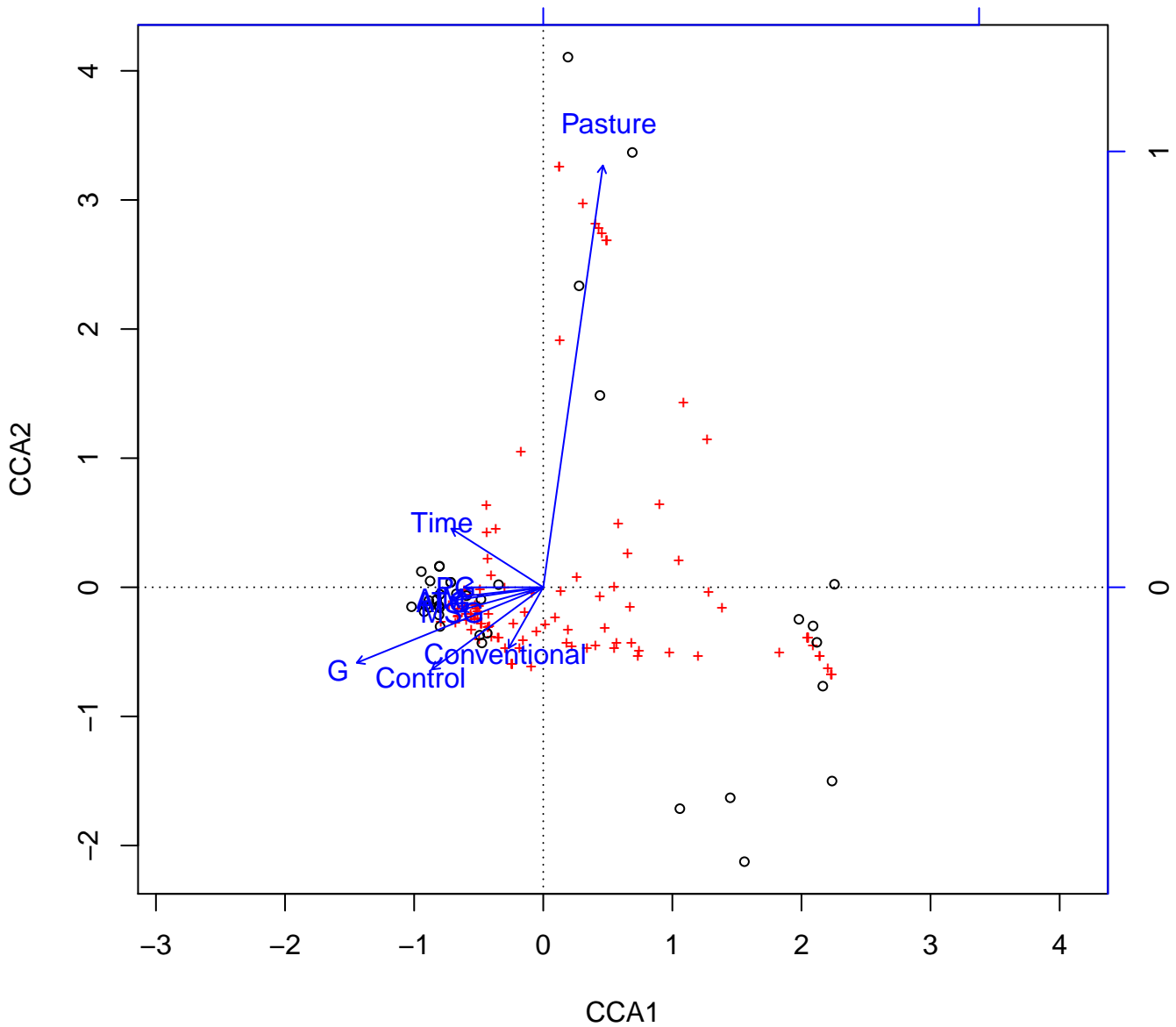

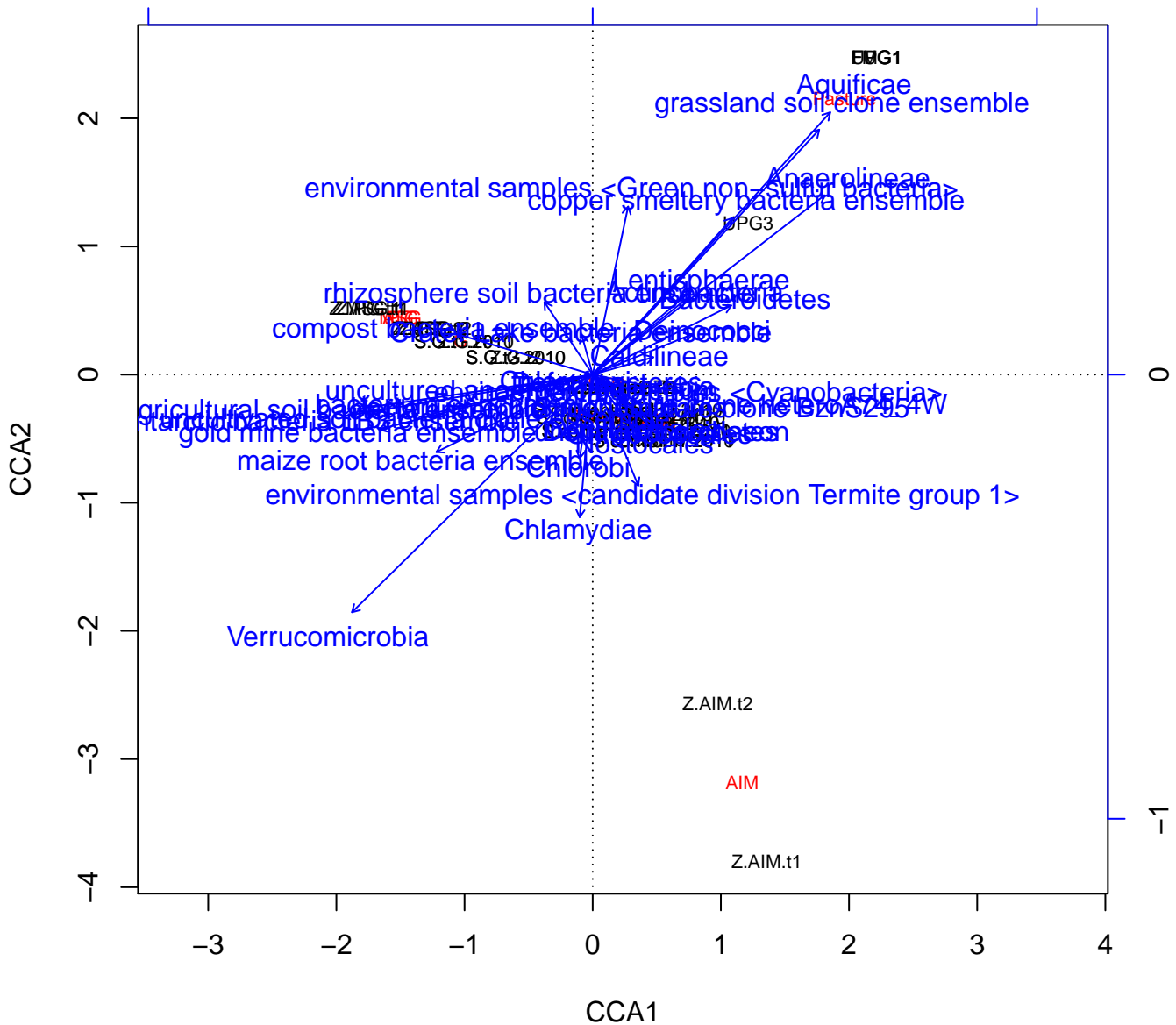

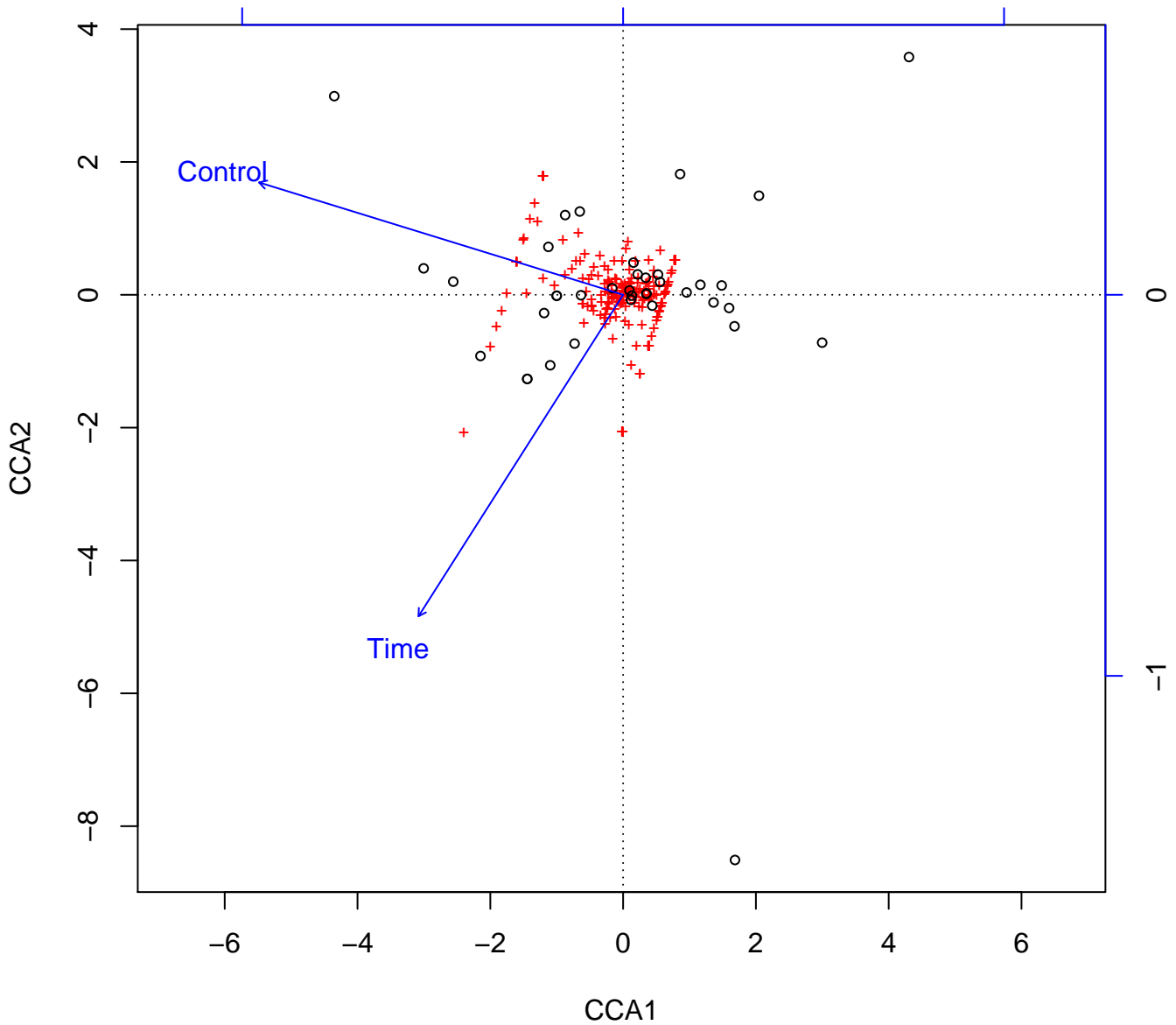

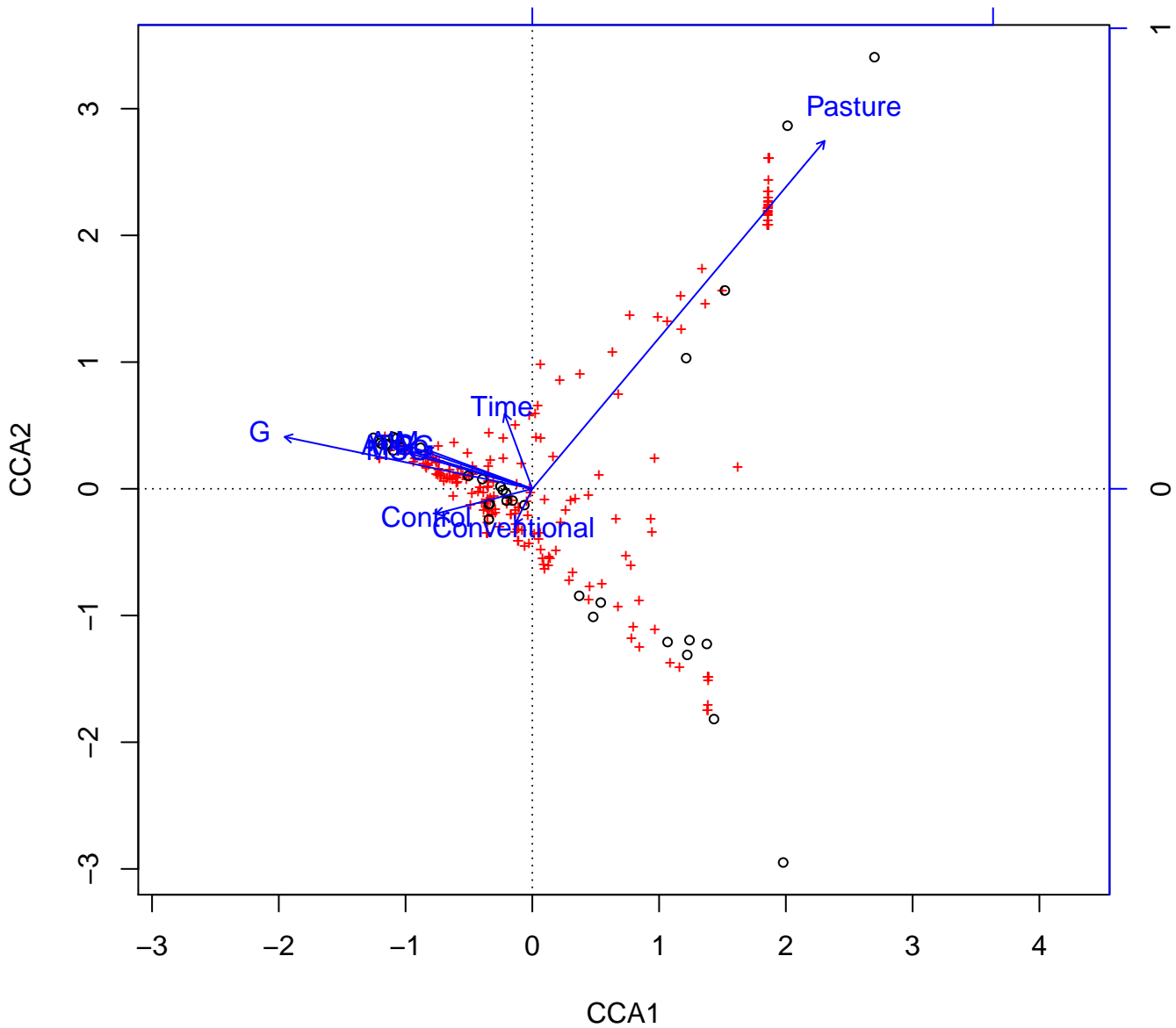



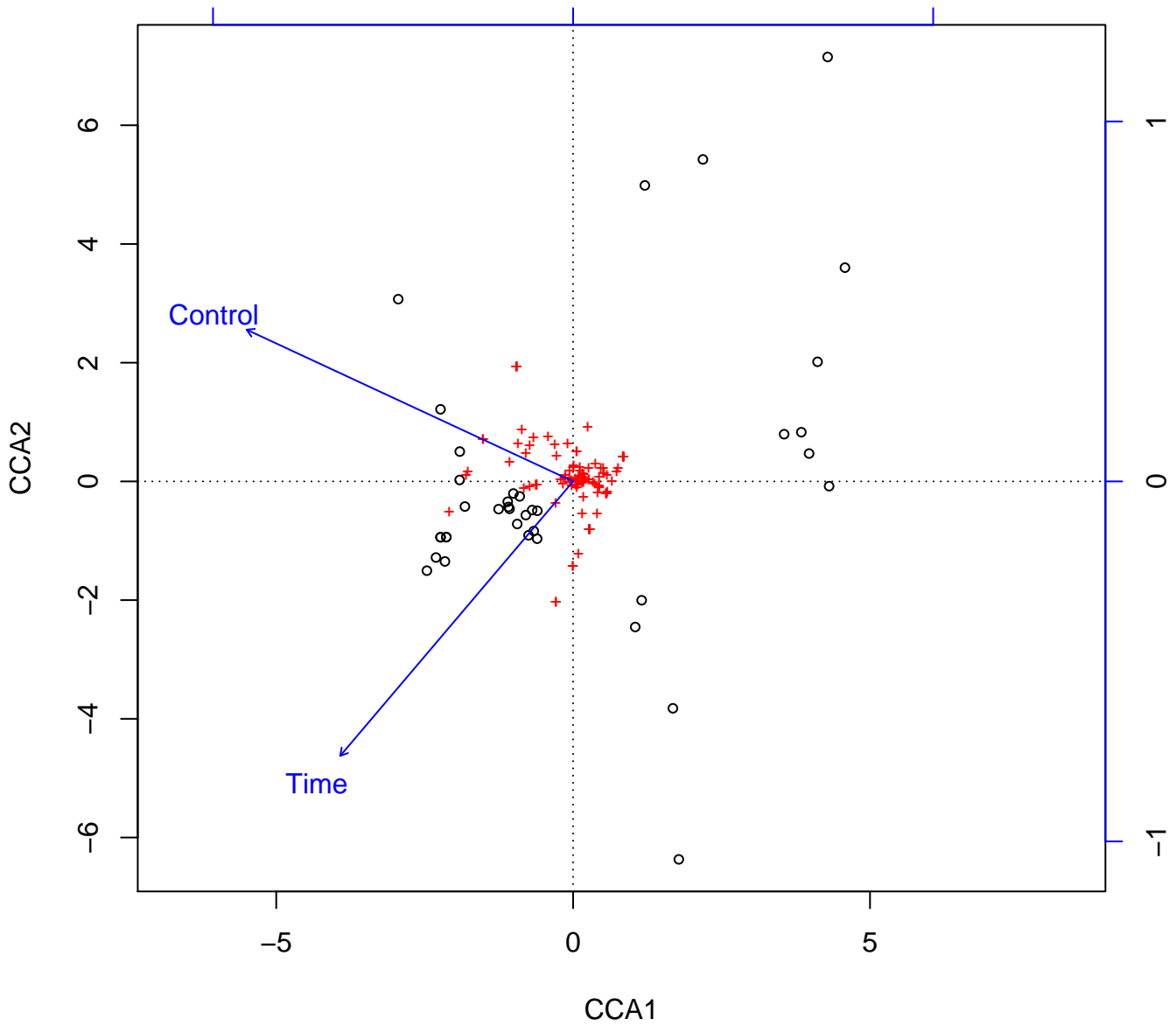

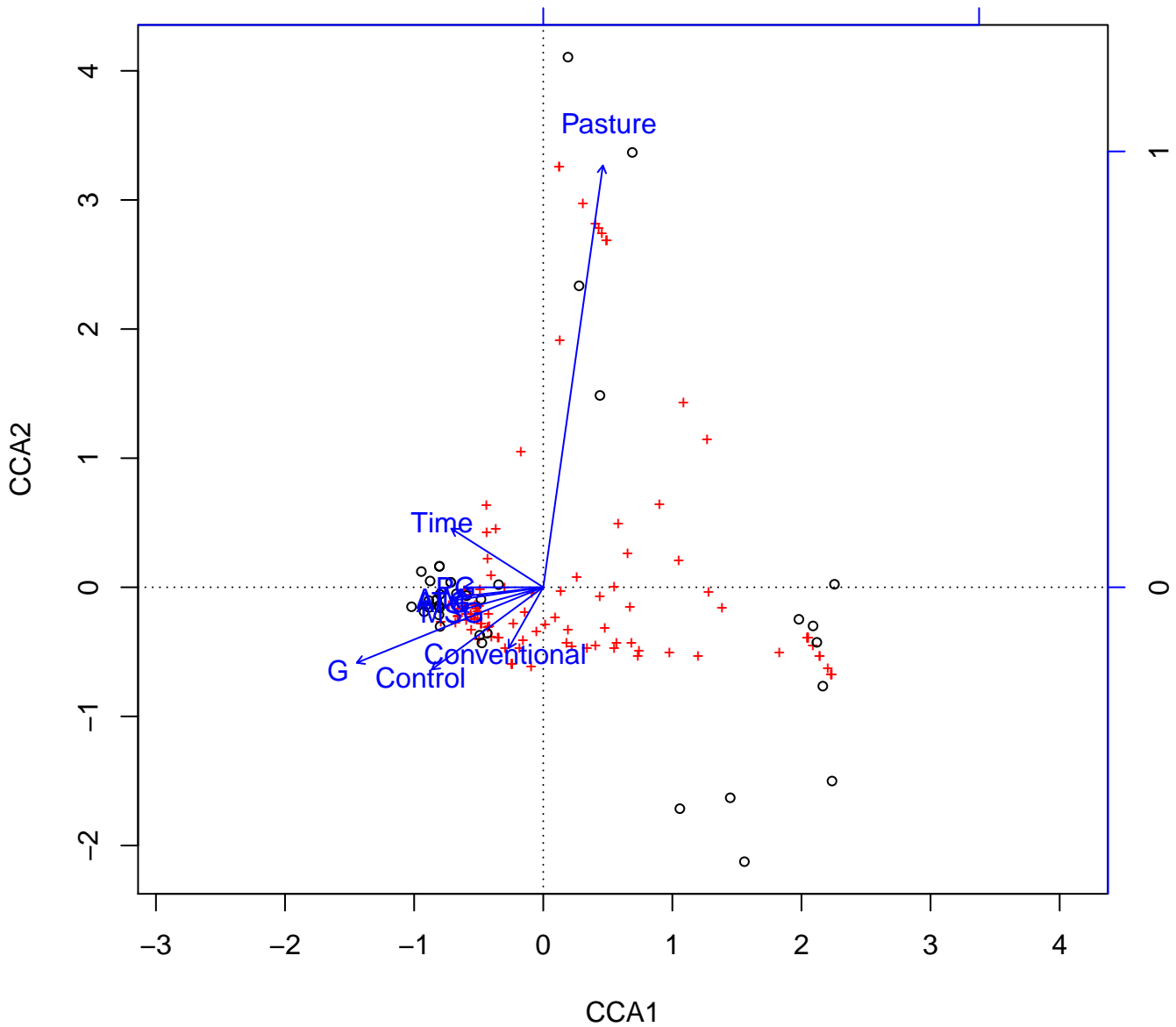

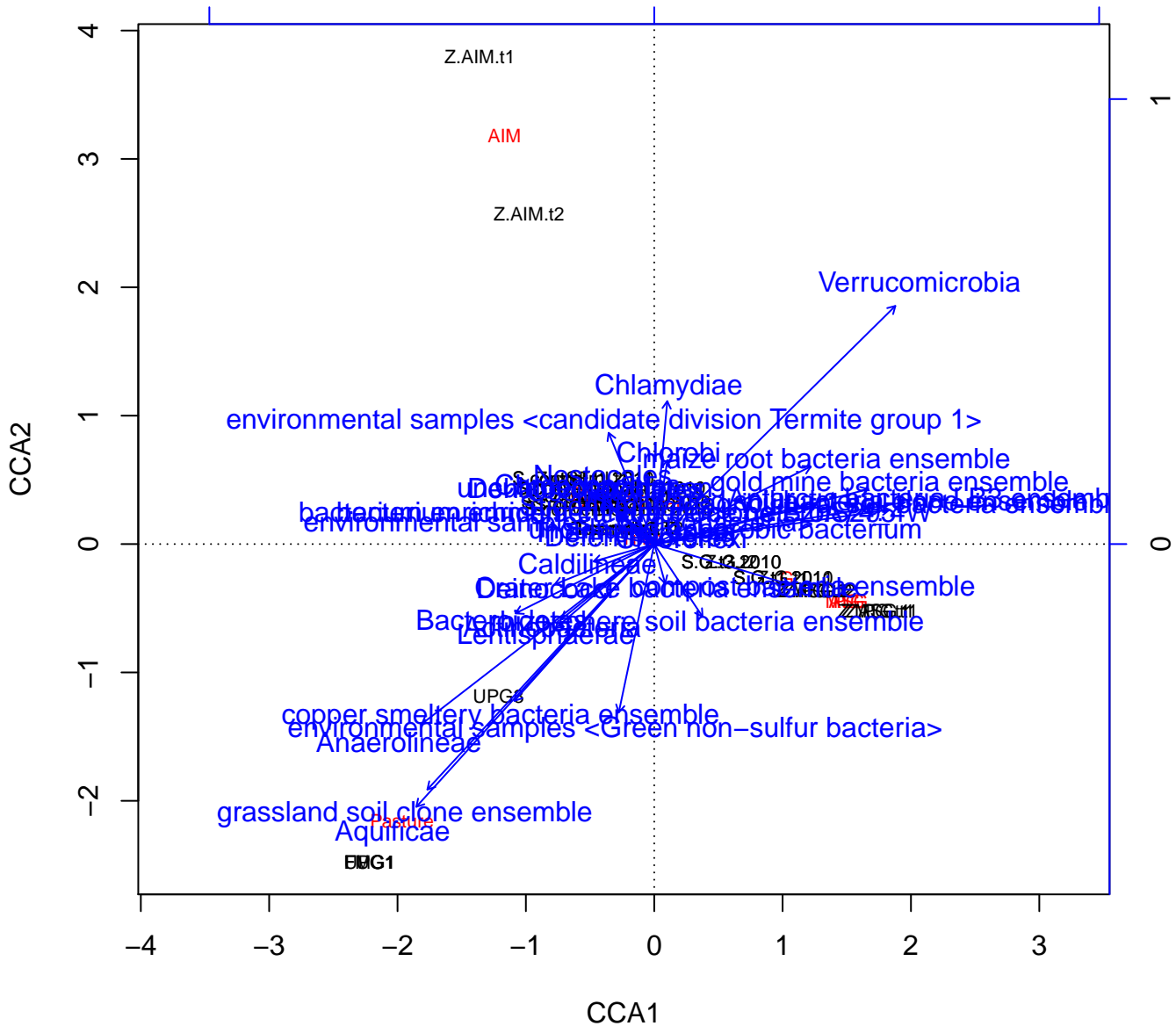

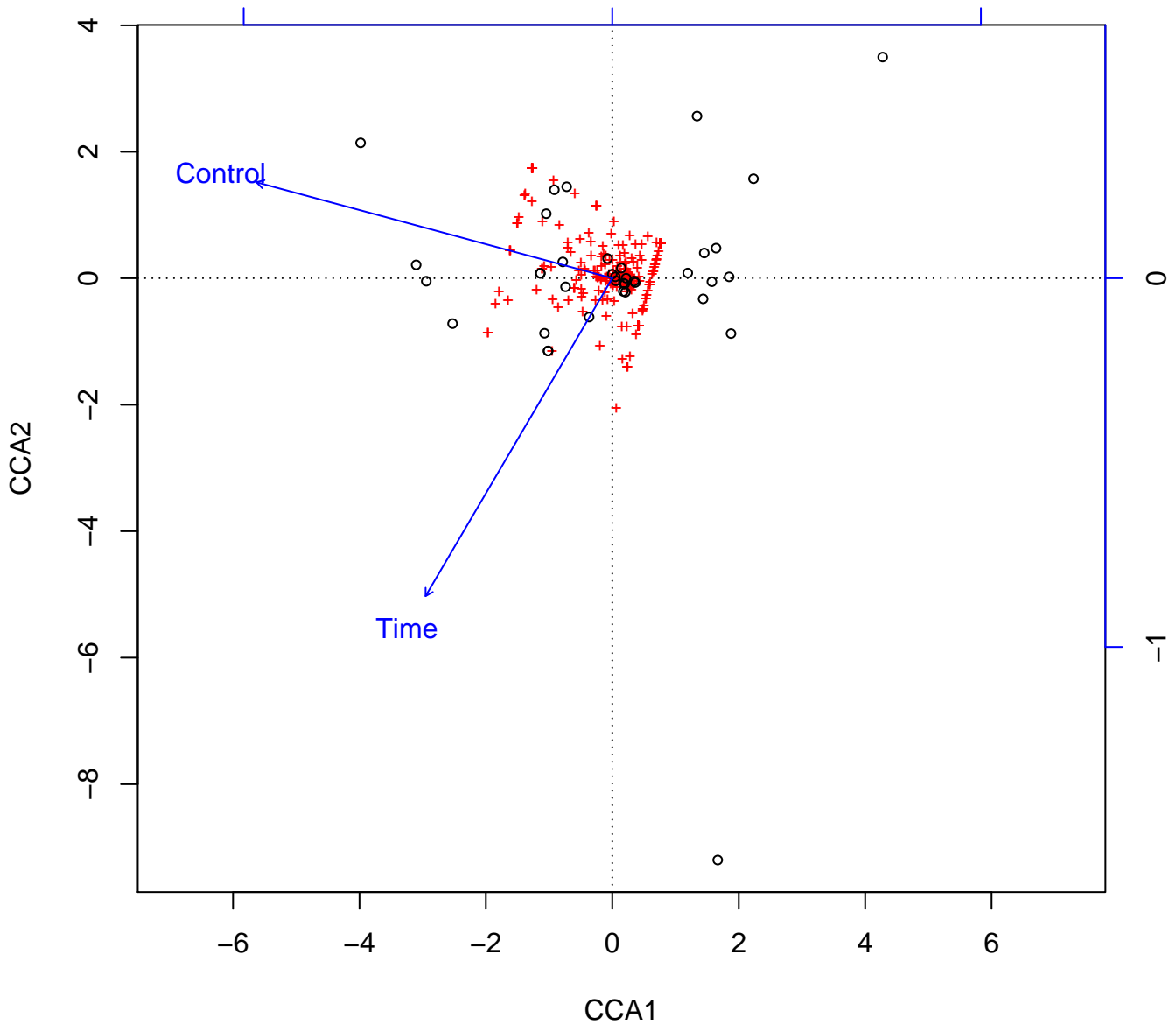

CCA2

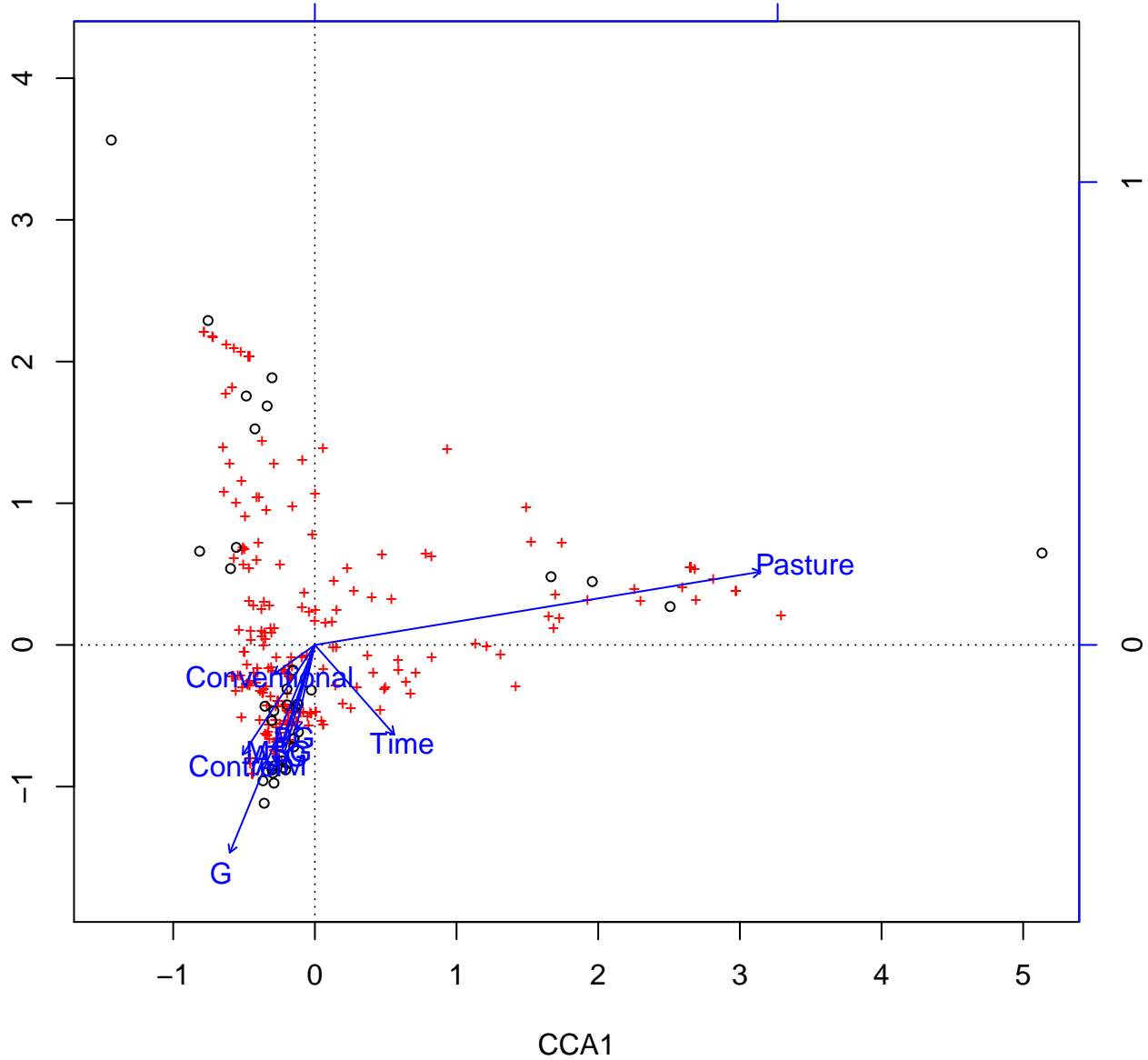

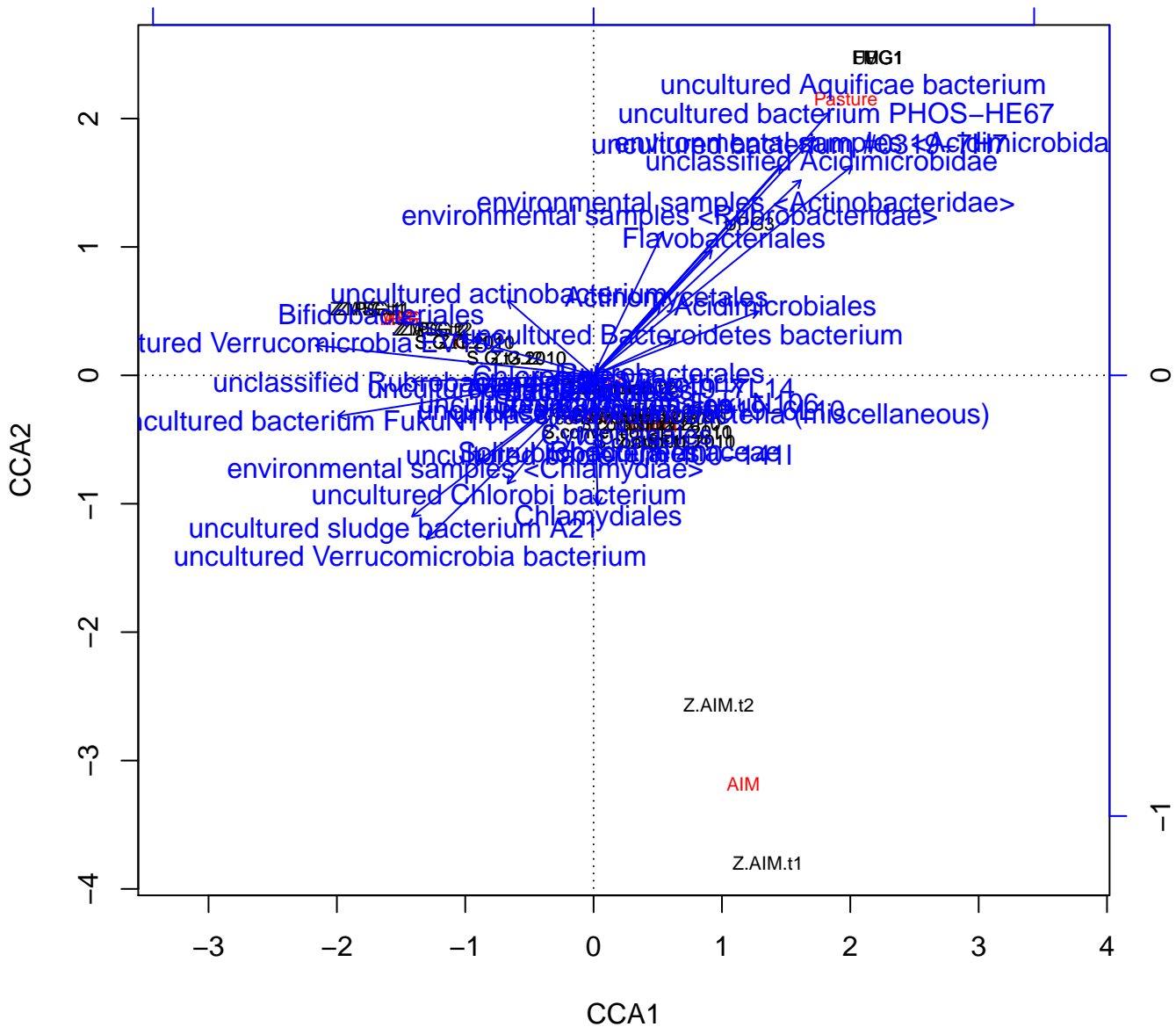

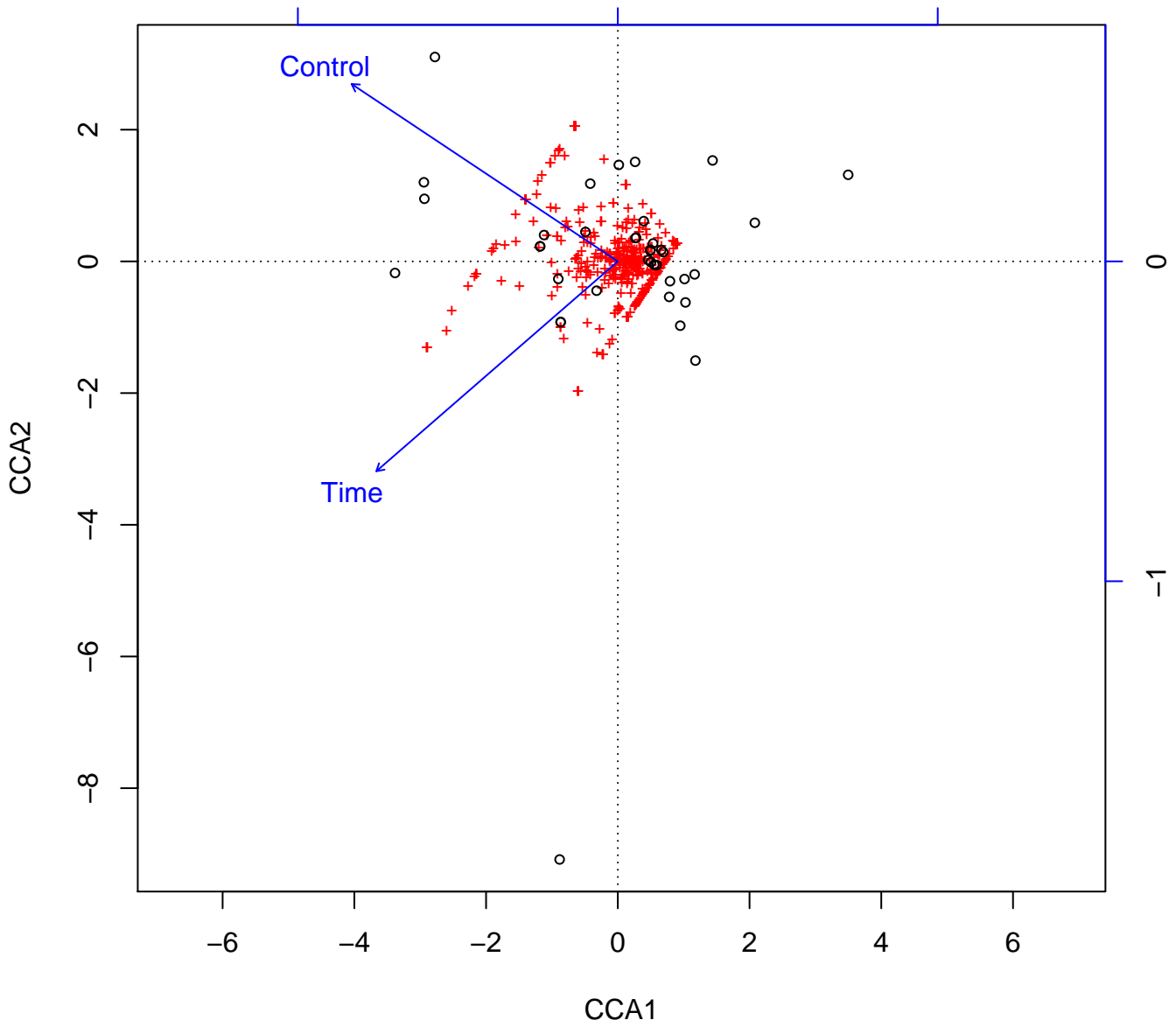

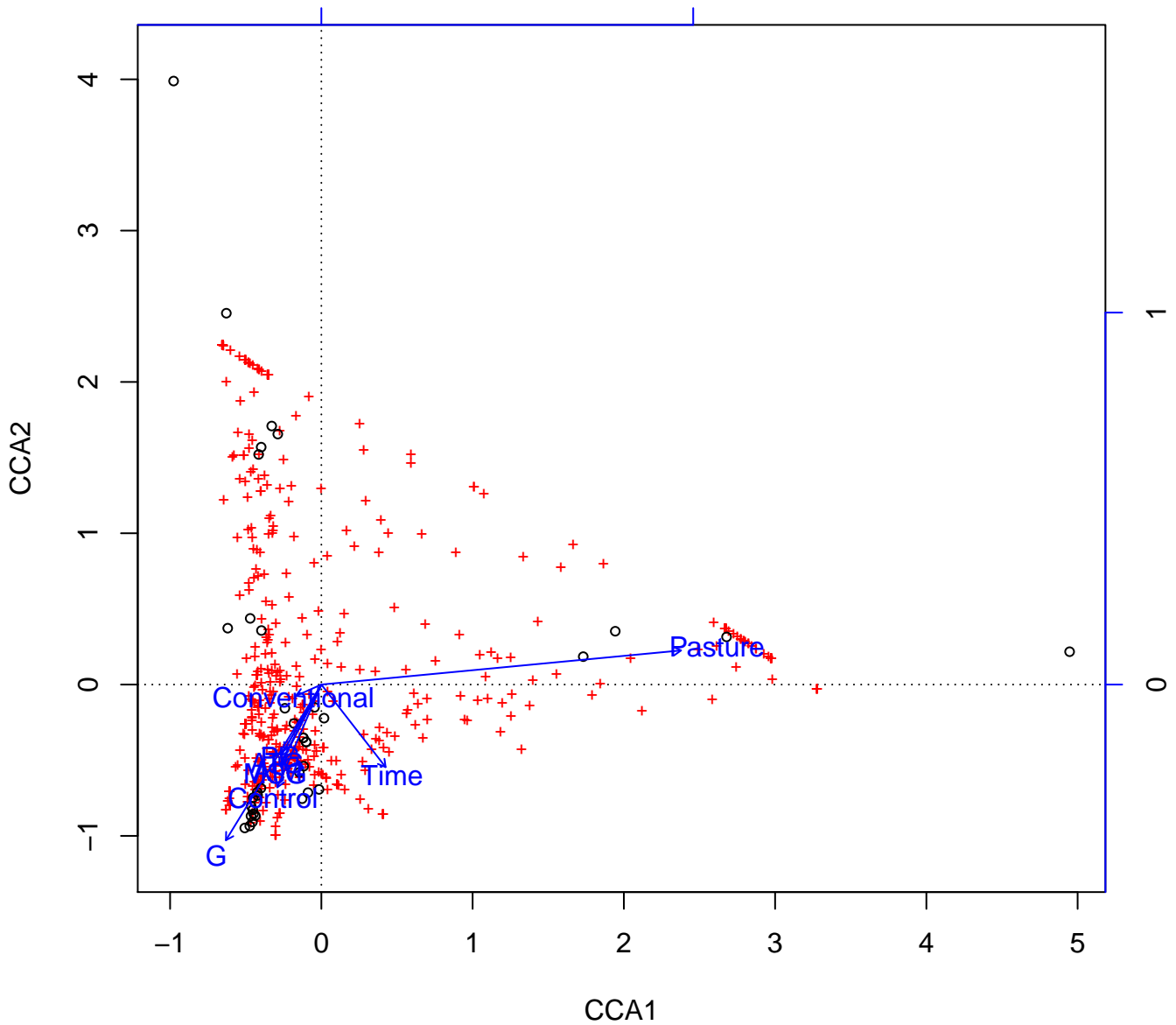

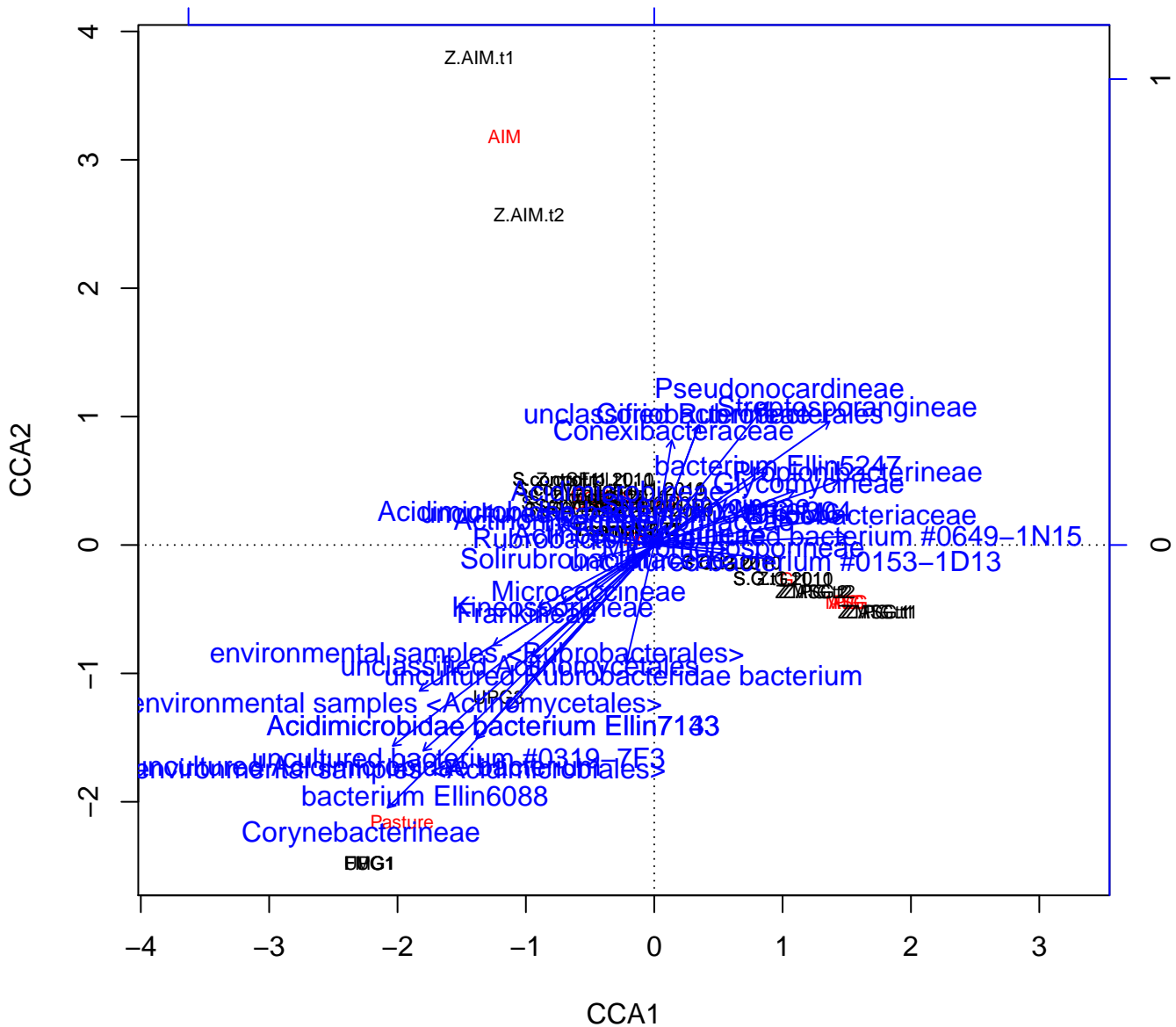

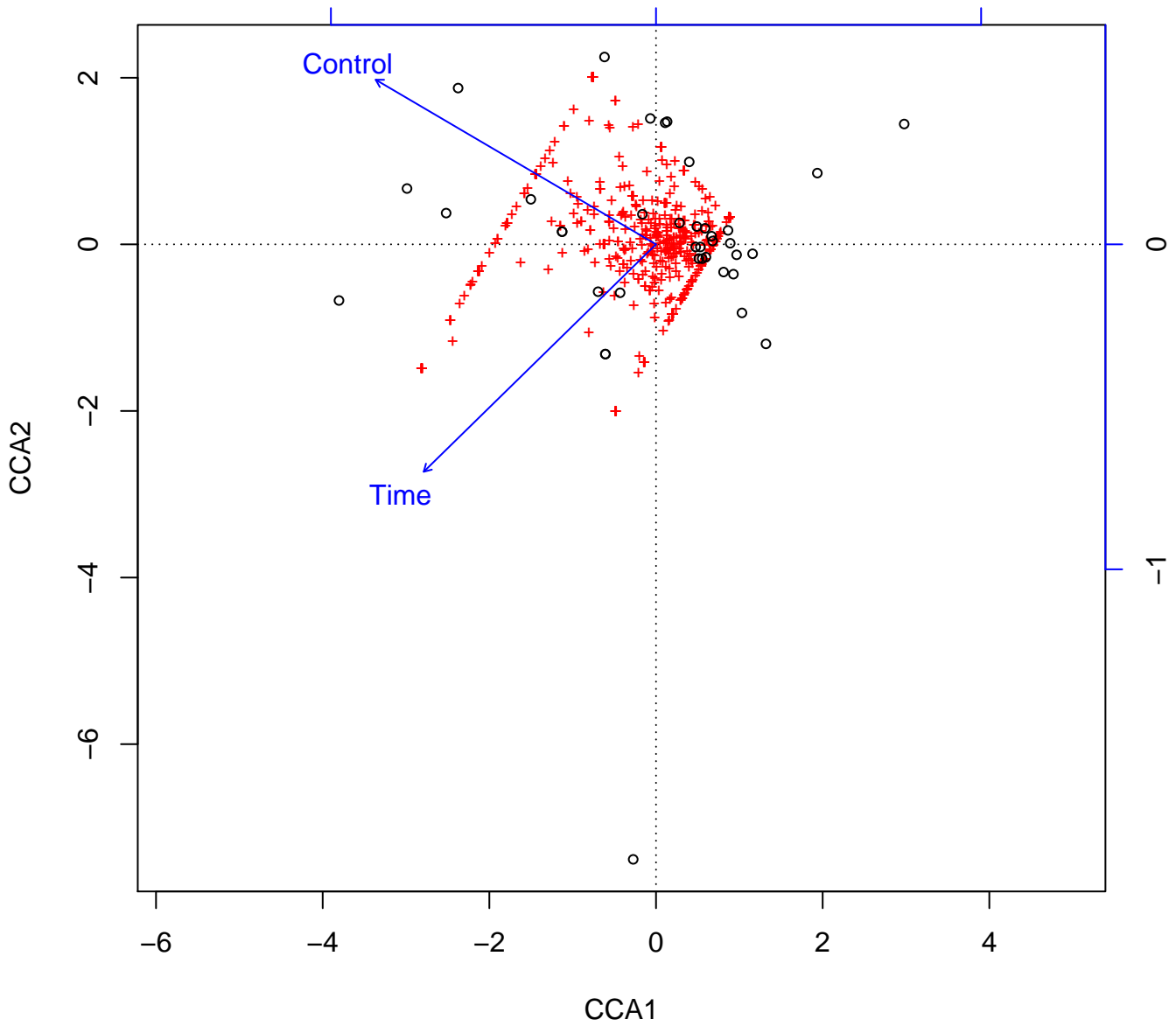

CCA2

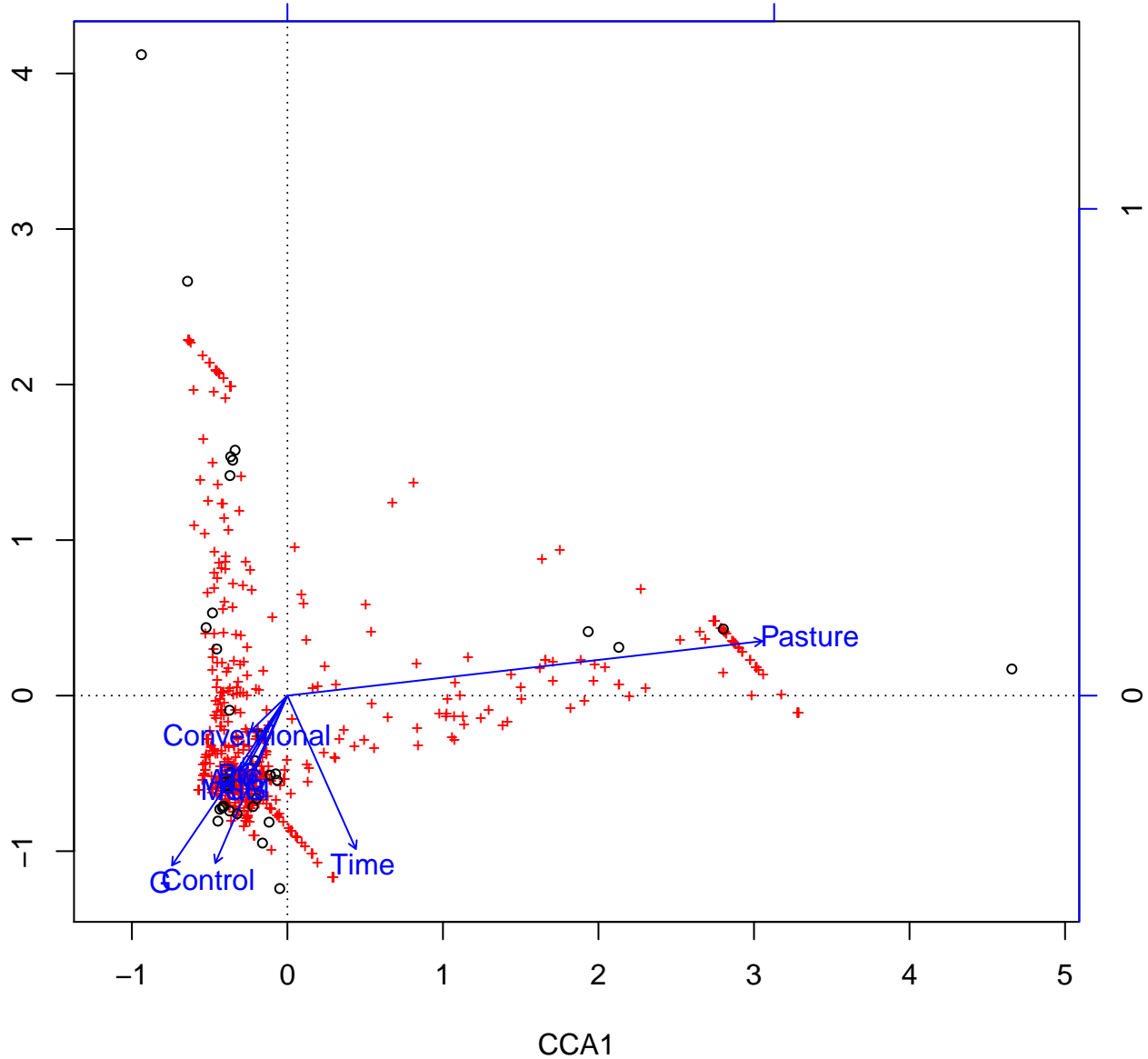

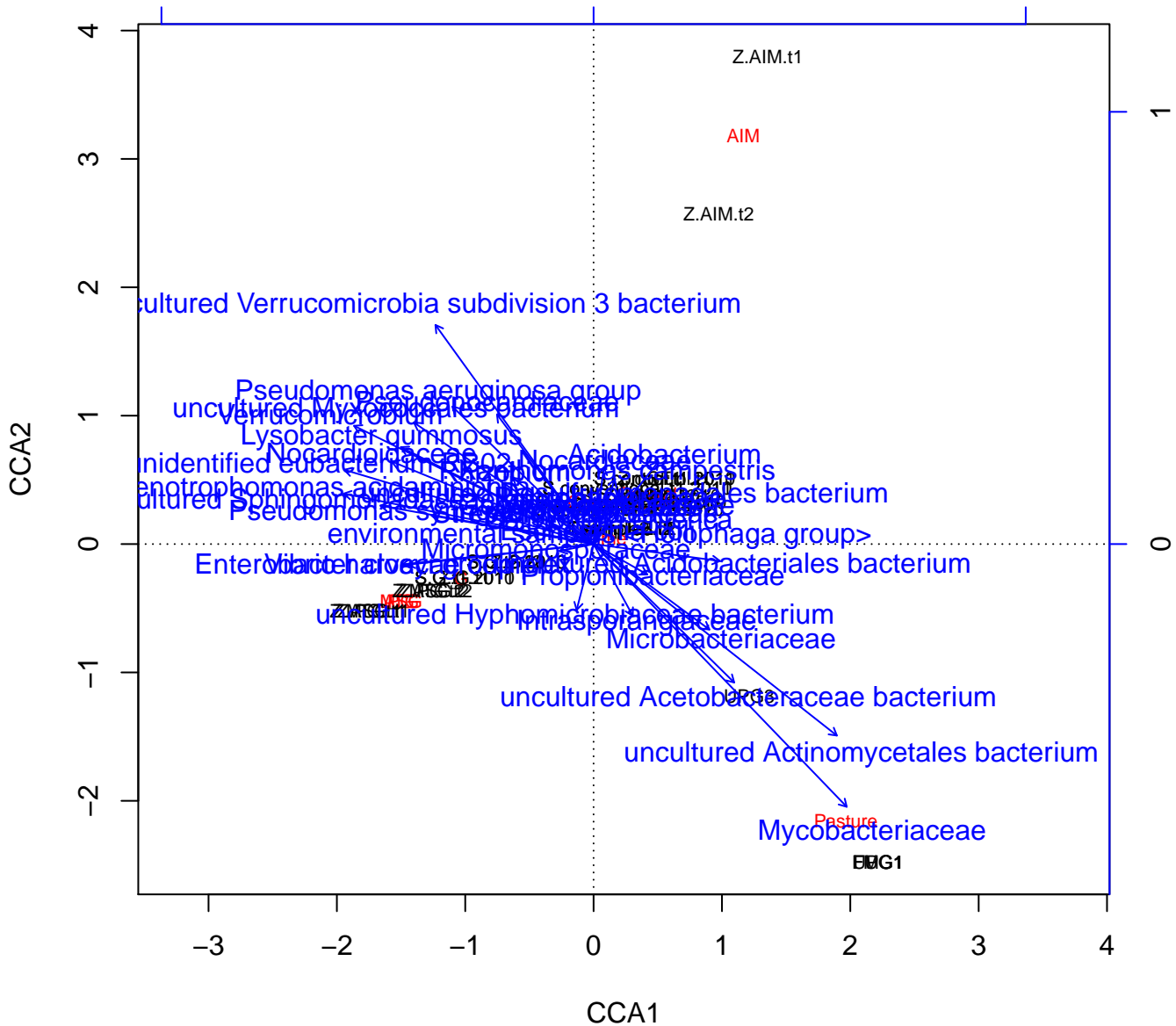

Supplement: S1 Appendix — The results of CCA and Indicator Value analyses obtained with each of the three classification methods are provided in separate subfolders as a compressed Zip archive. (ZIP) [file pone.0165204.s001.zip › S2_folder_R_analyses/megan/cca/megan_CCA_R_plots.pdf]

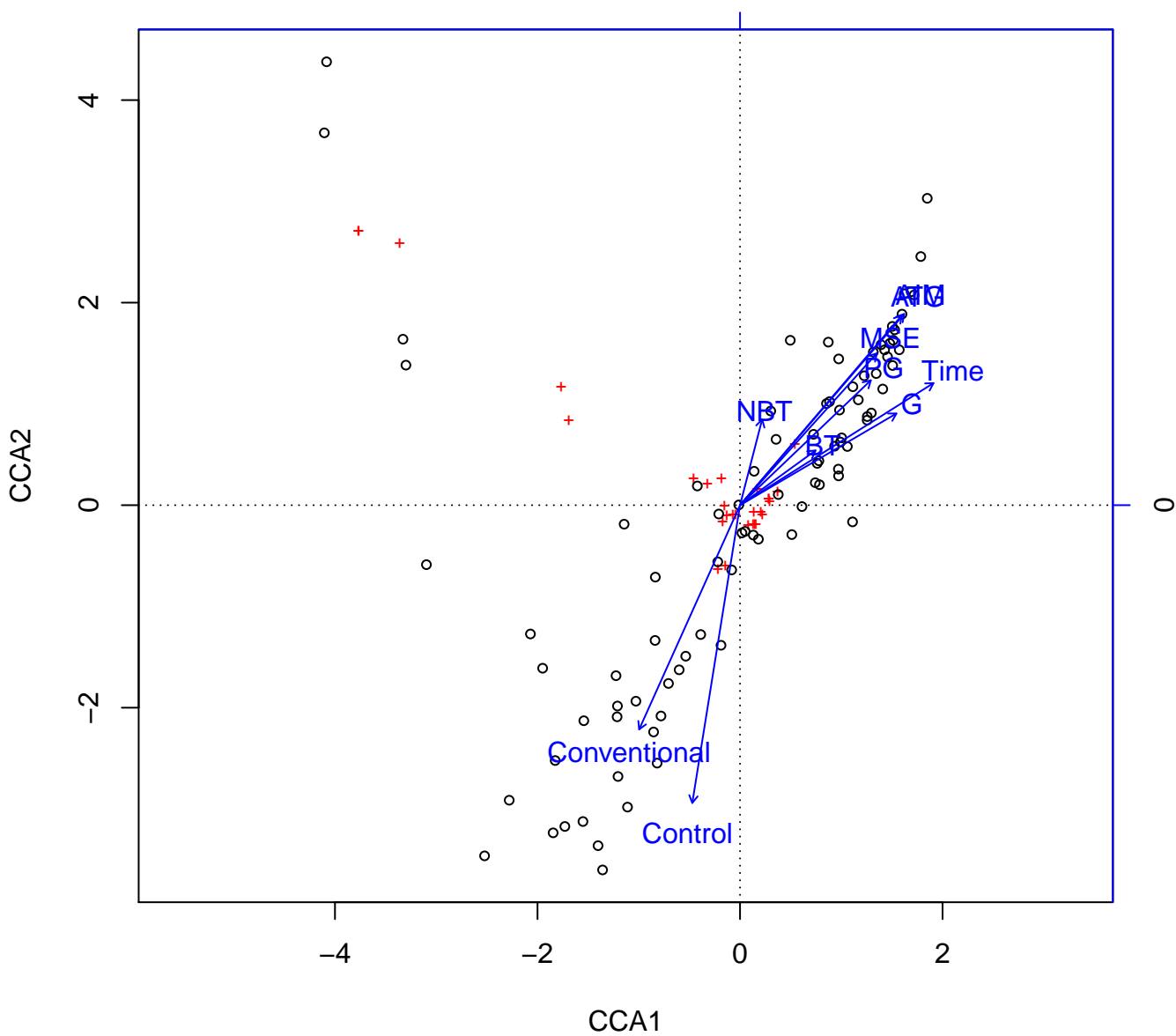

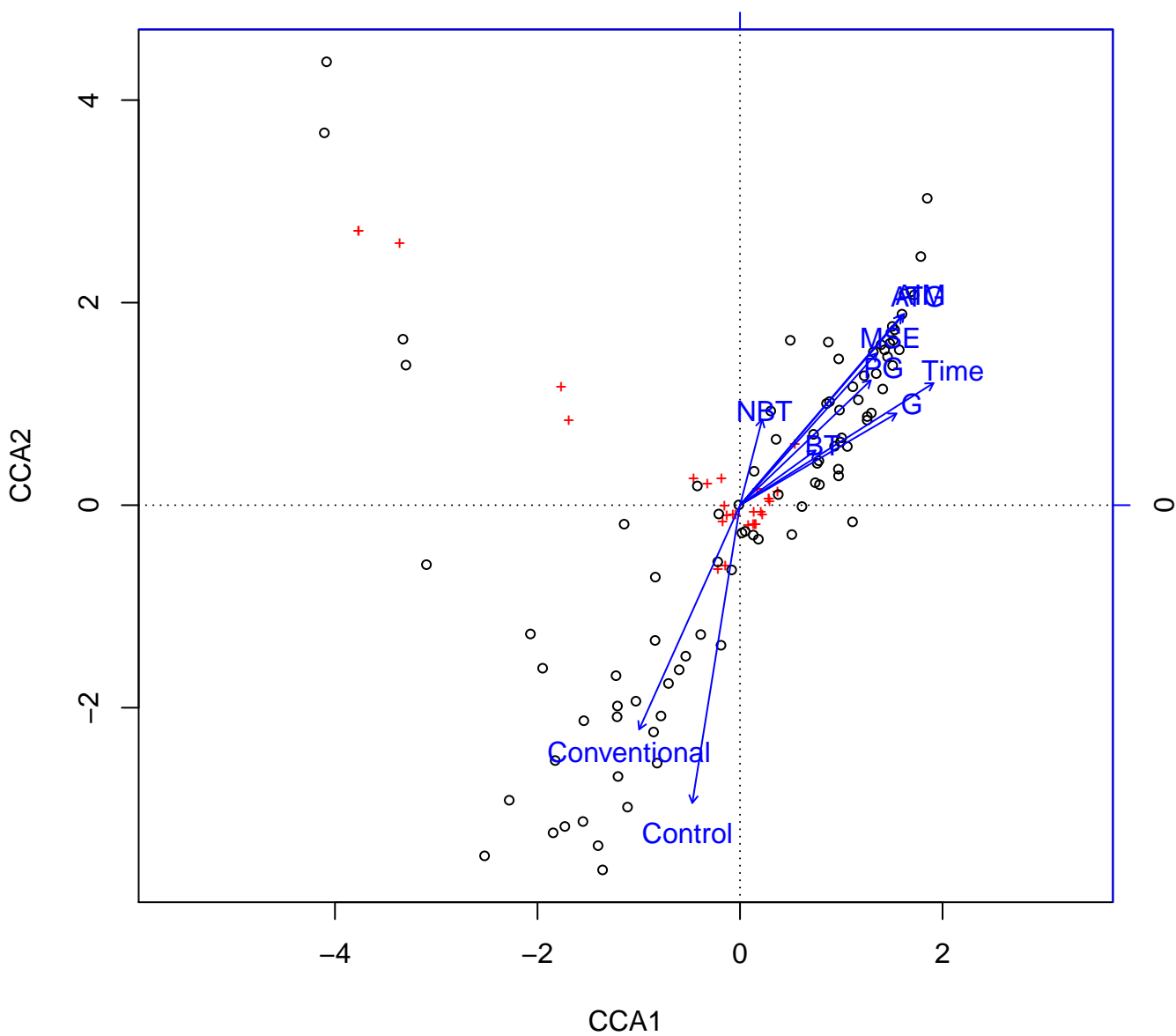

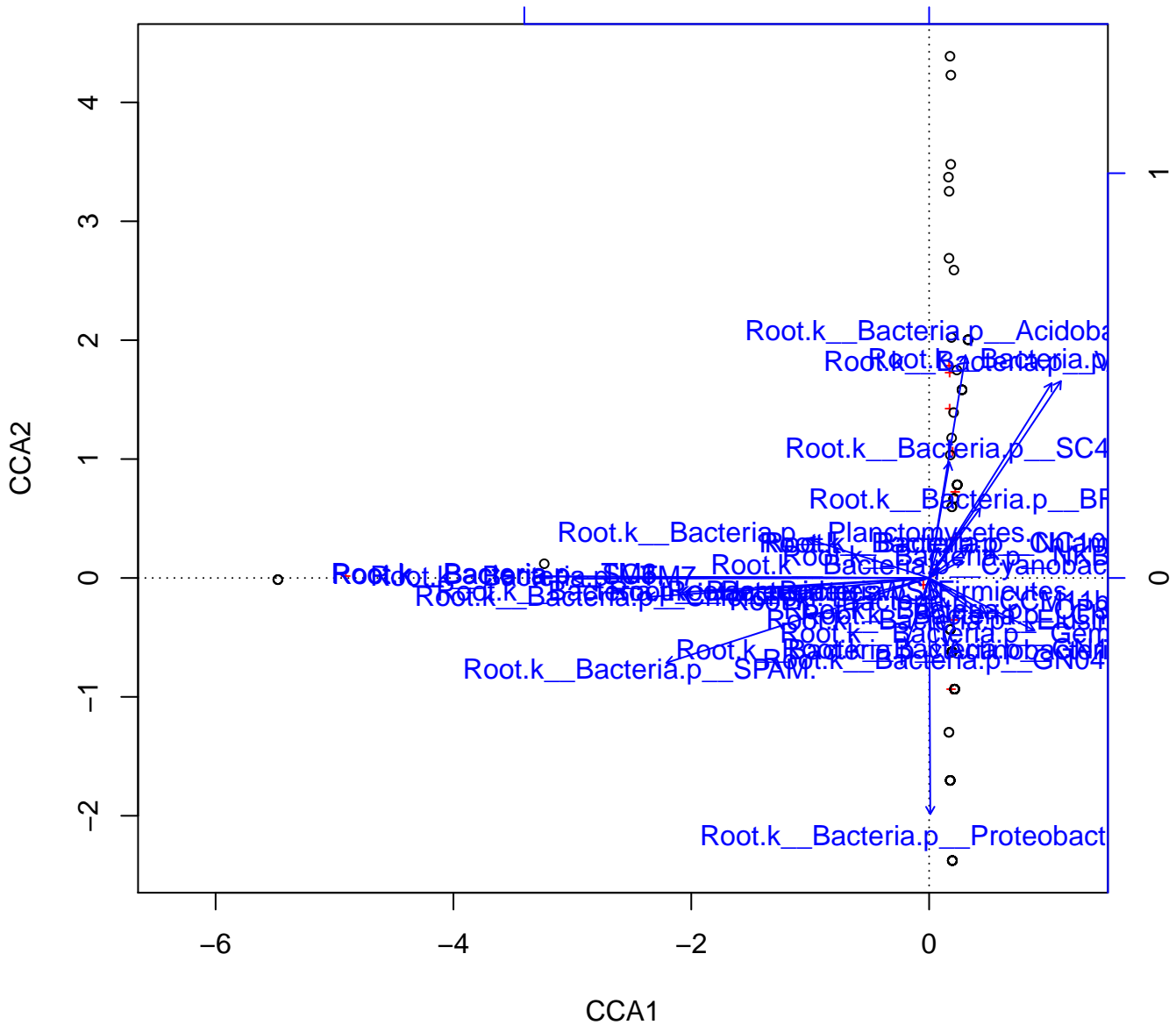

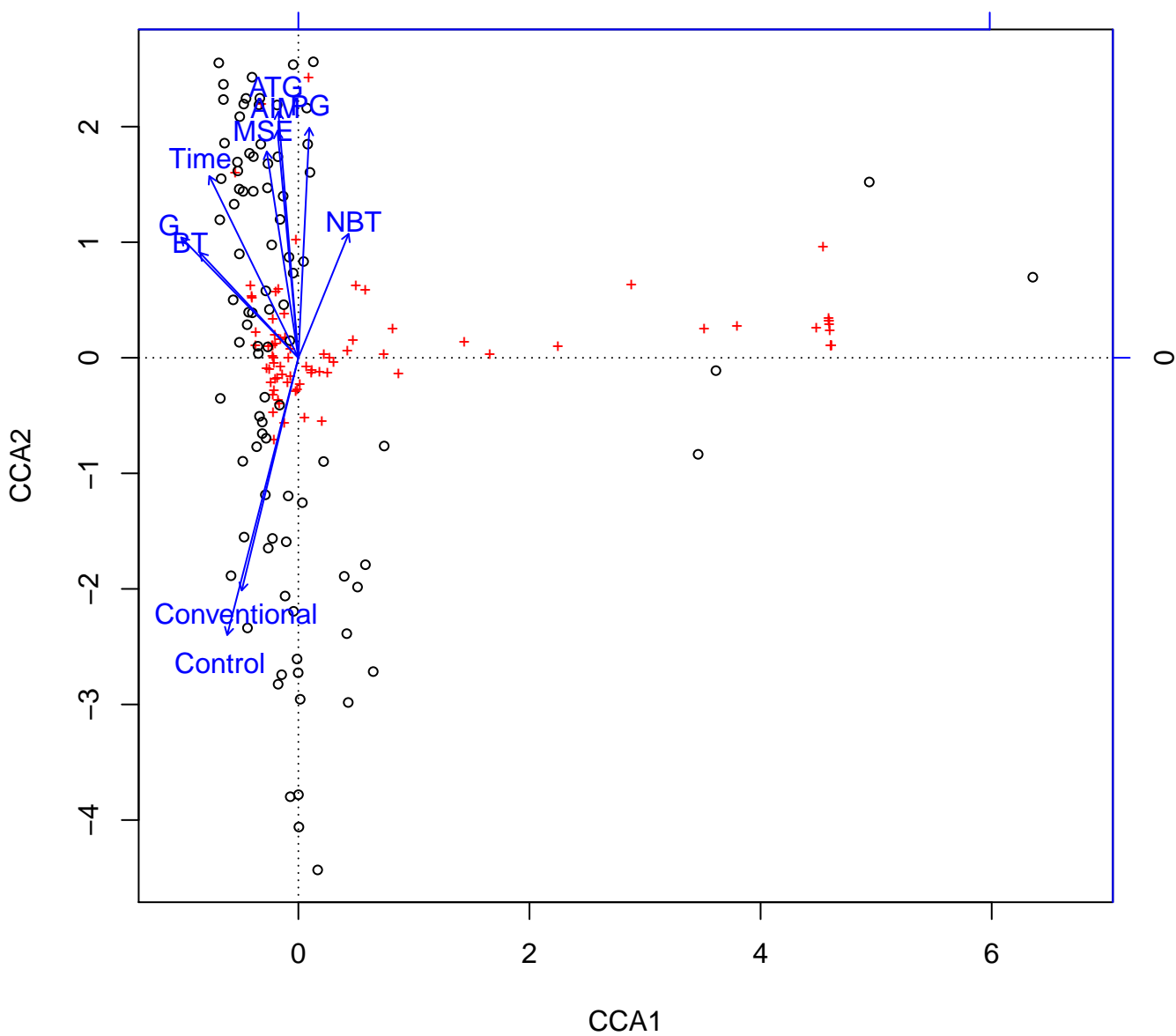

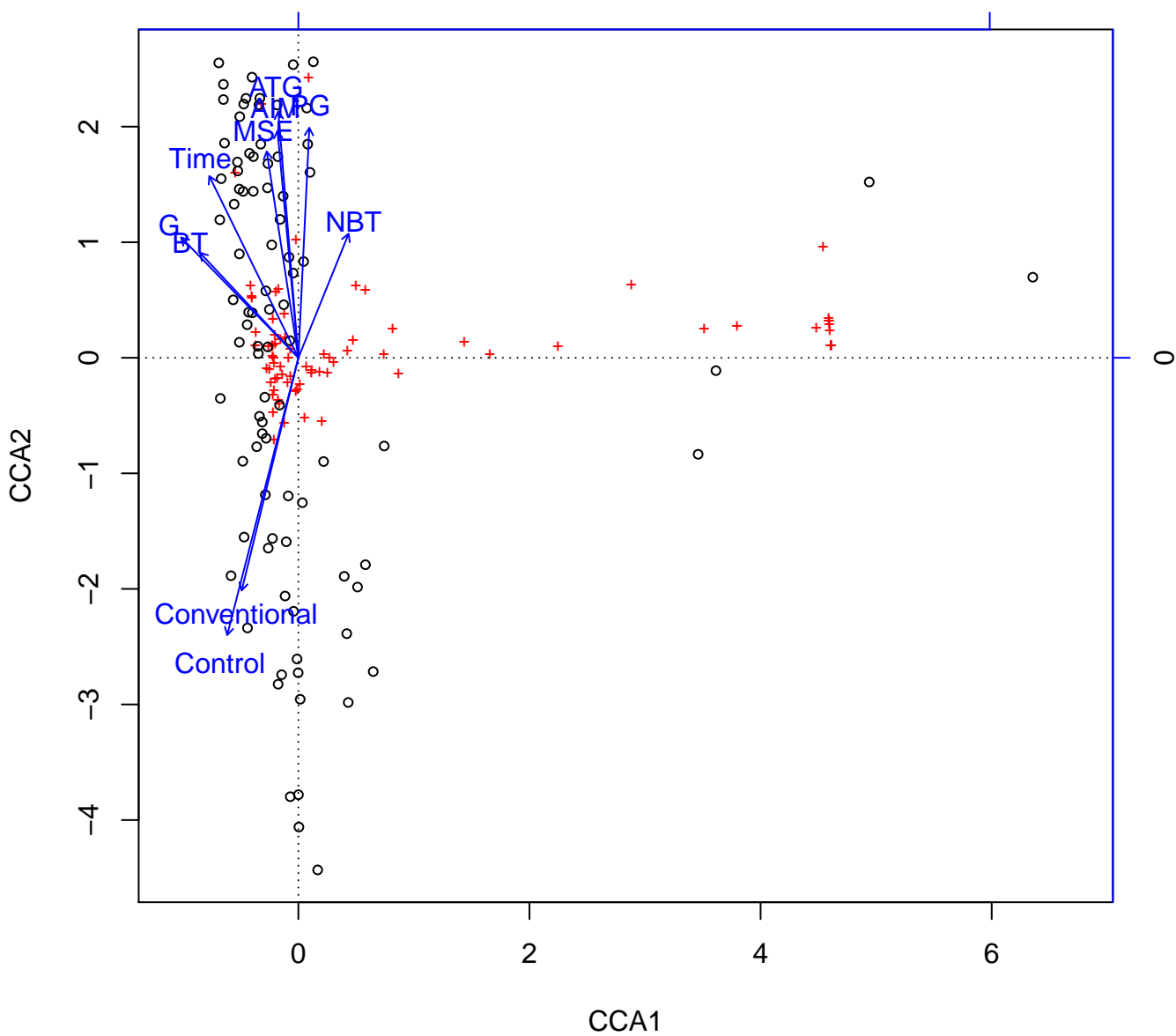

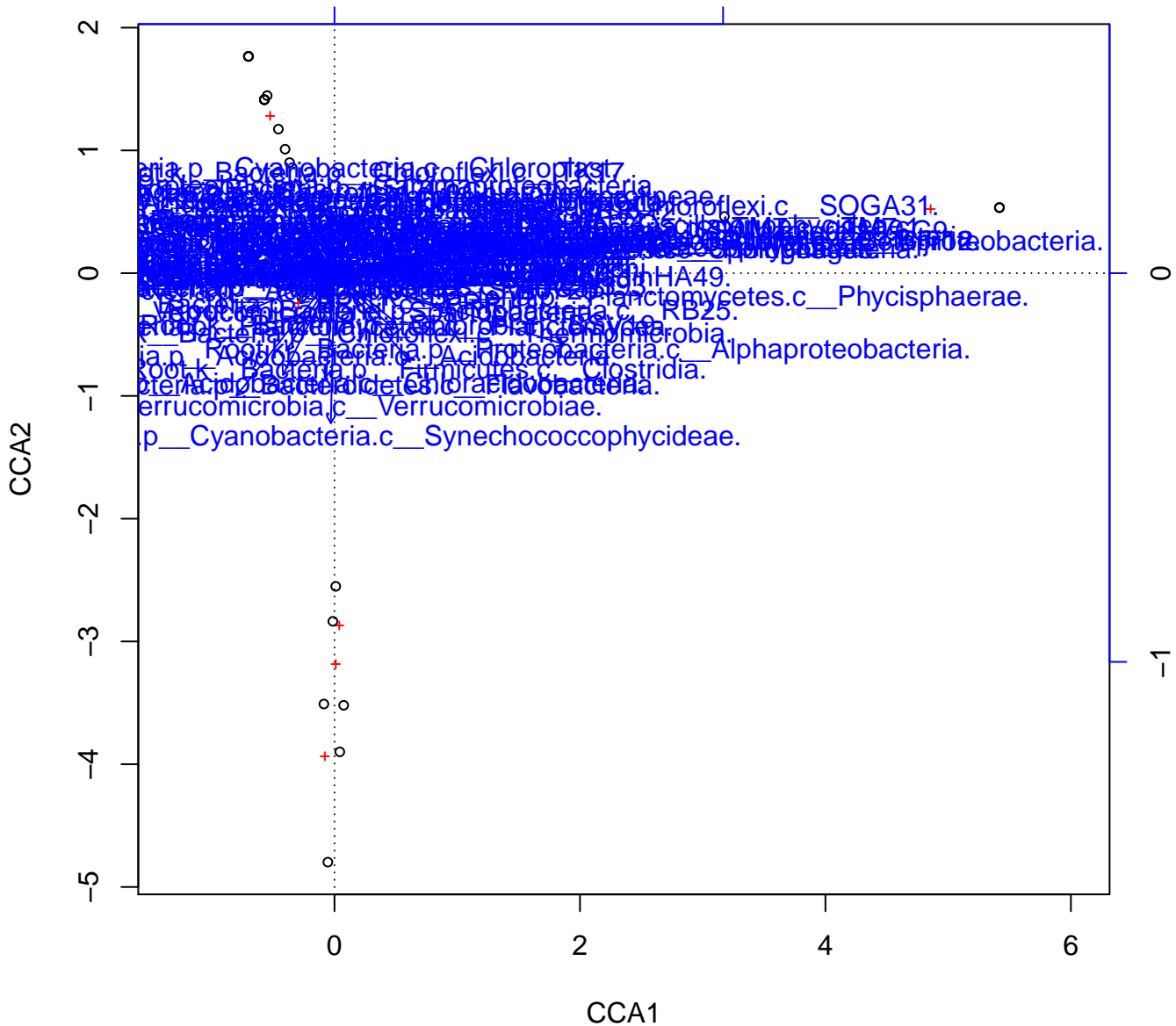

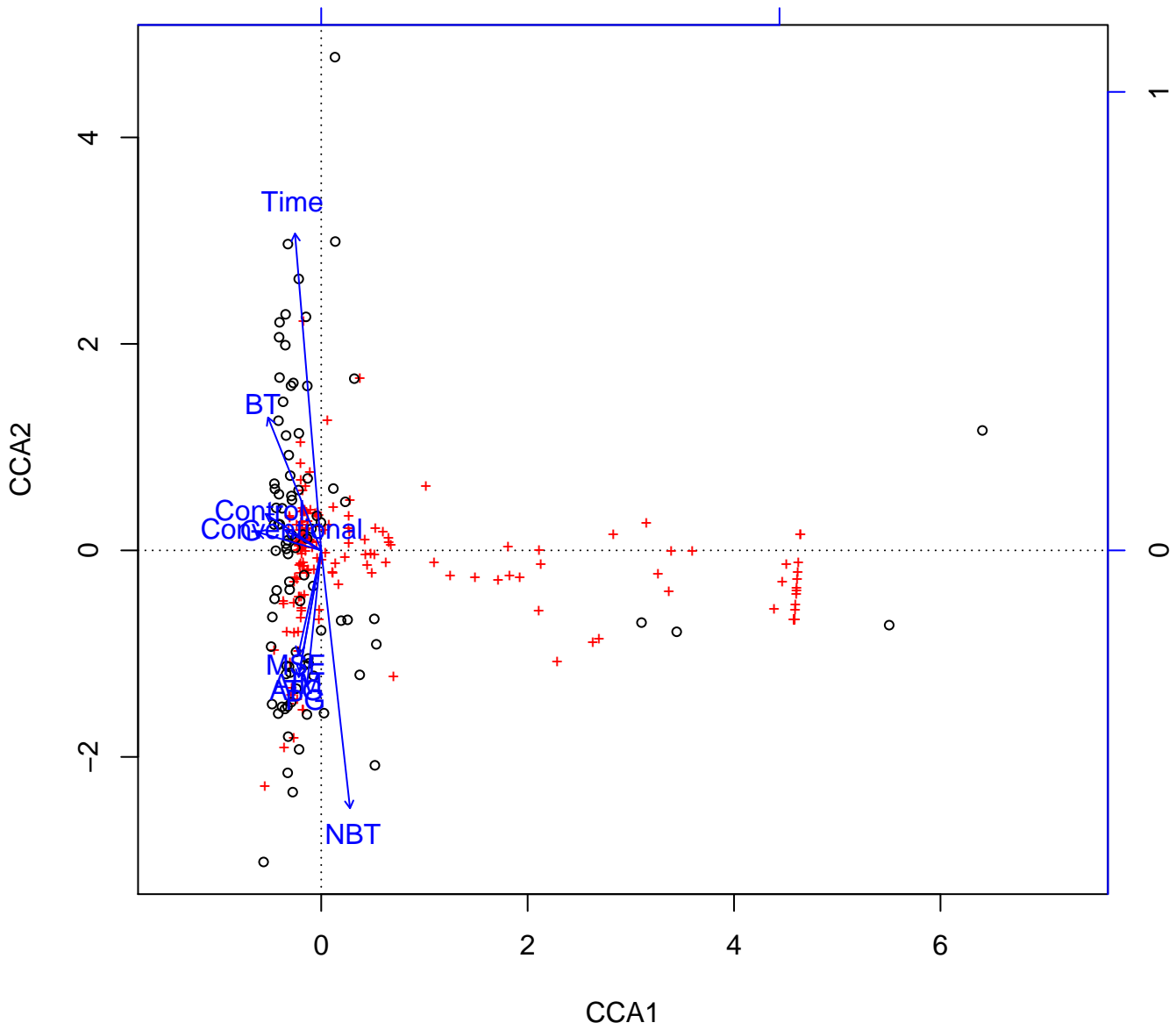

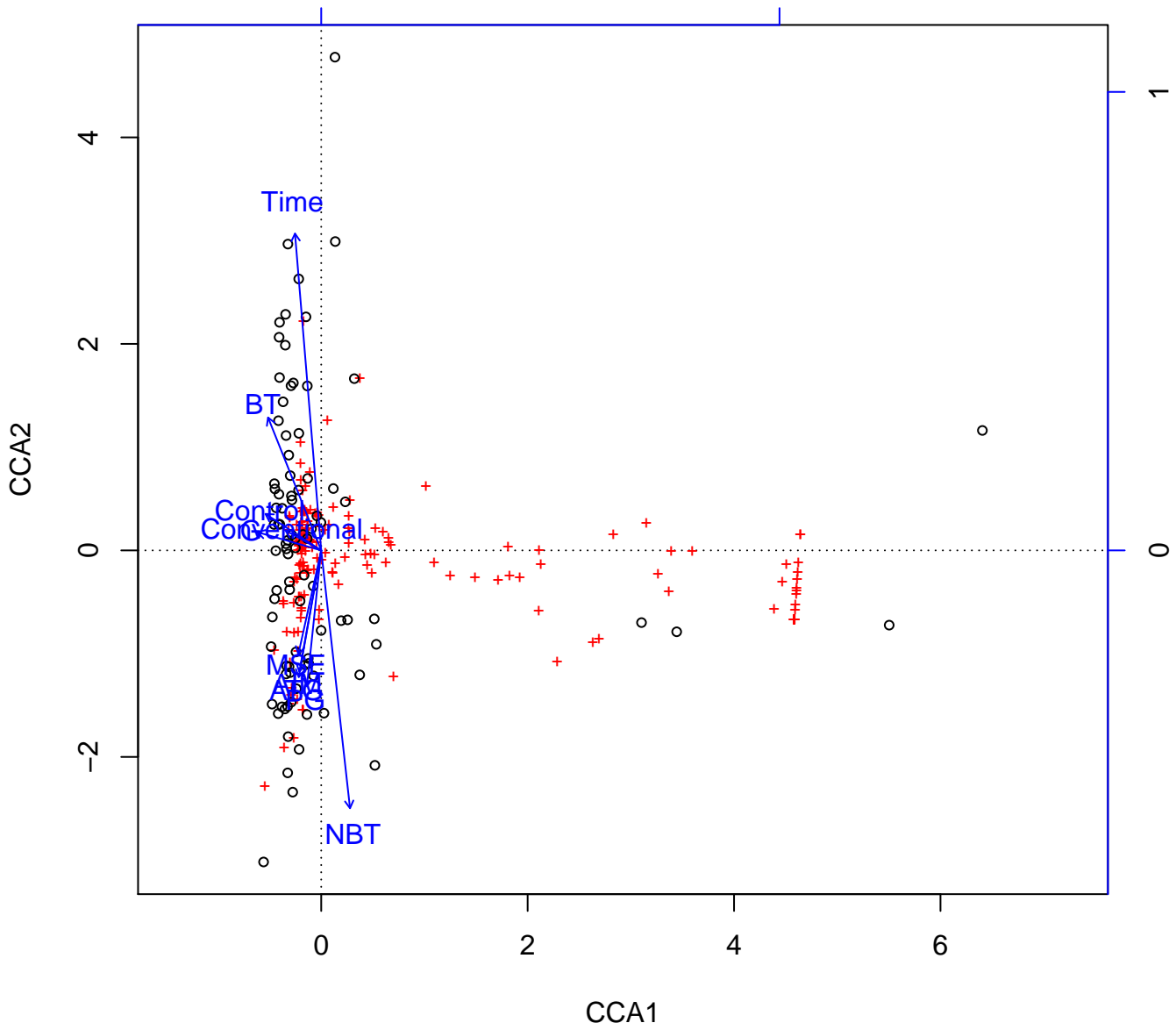

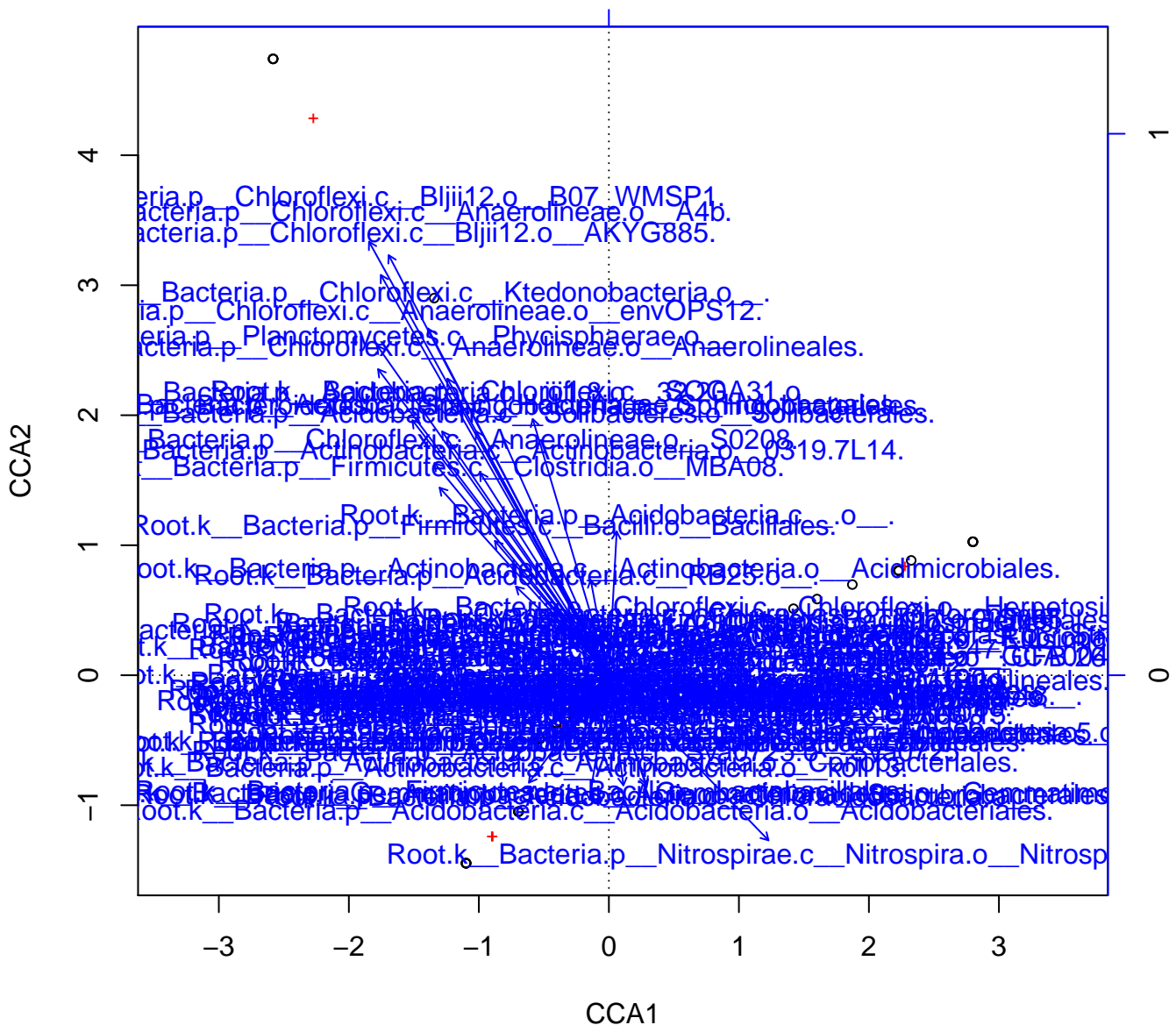

Supplement: S1 Appendix — The results of CCA and Indicator Value analyses obtained with each of the three classification methods are provided in separate subfolders as a compressed Zip archive. (ZIP) [file pone.0165204.s001.zip › S2_folder_R_analyses/rdp/cca/rdp_CCA_R_plots.pdf]

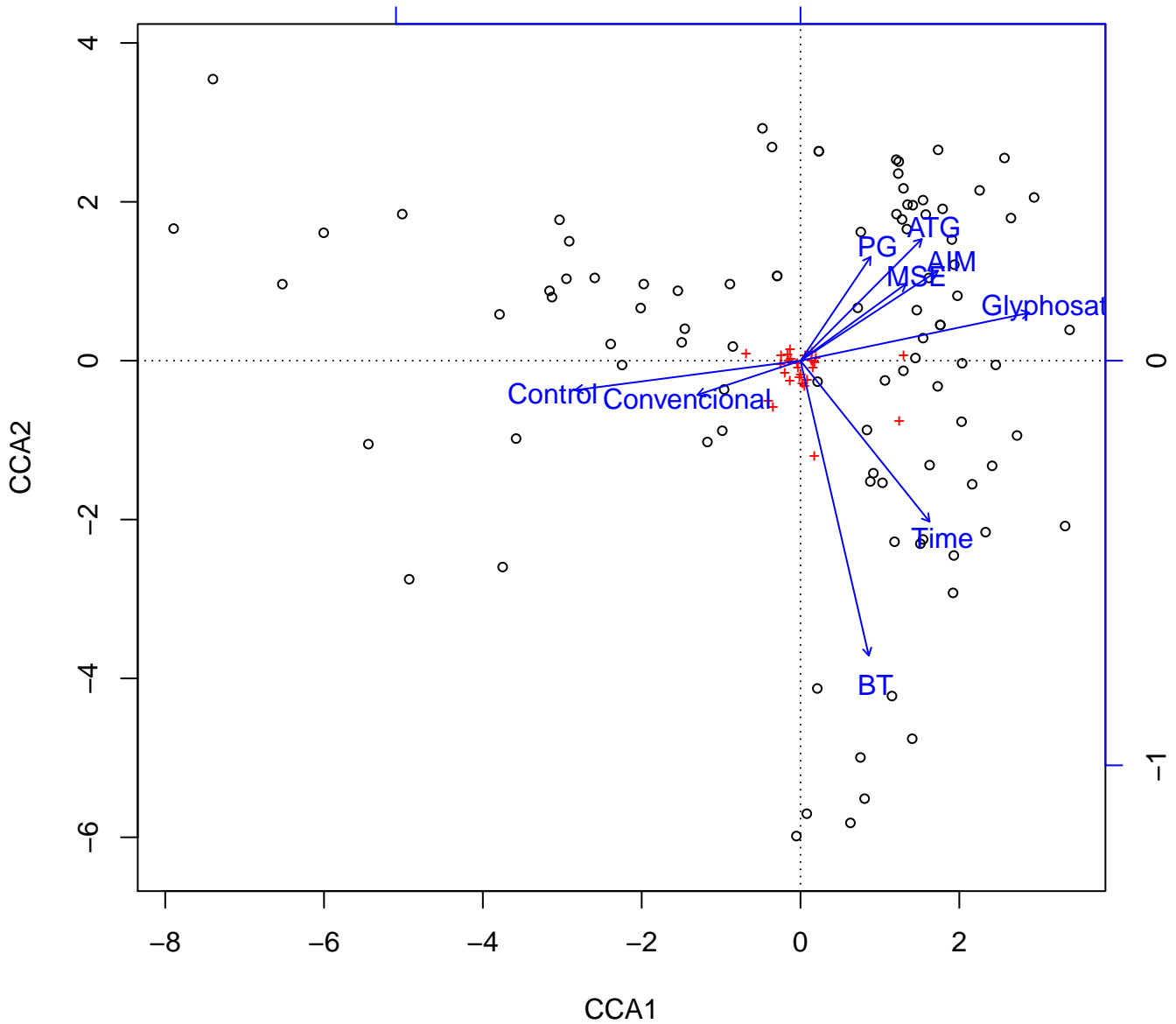

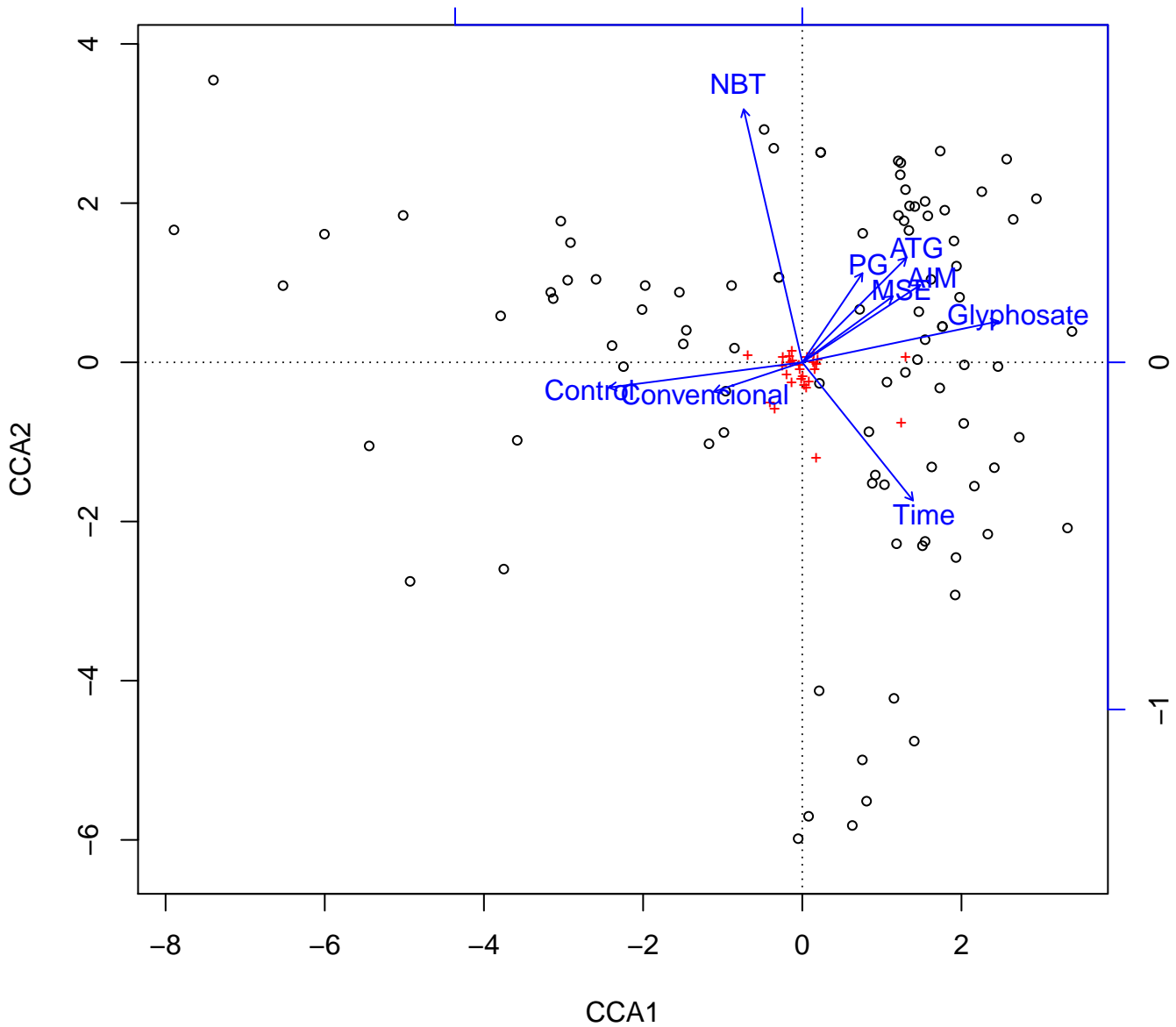

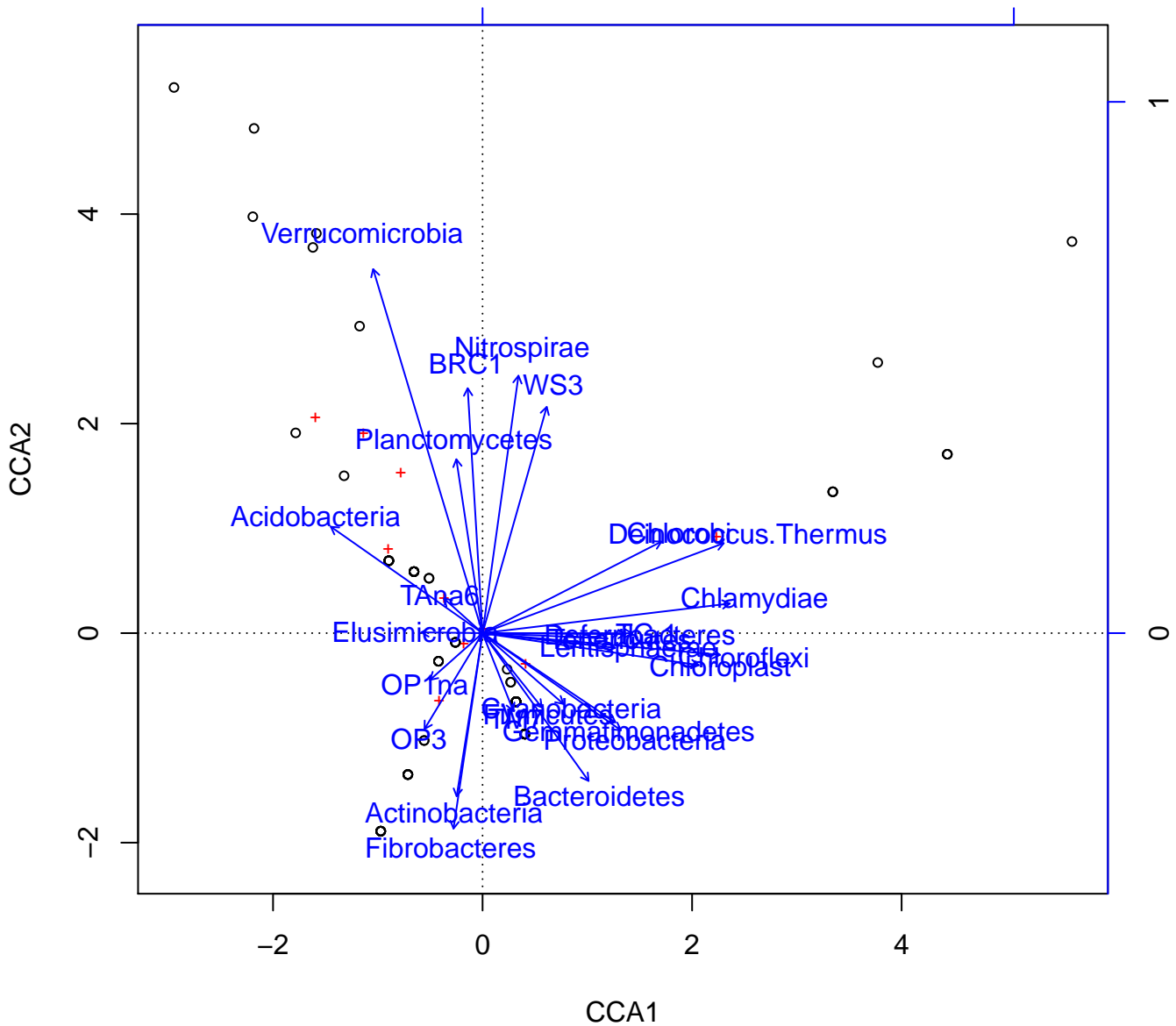

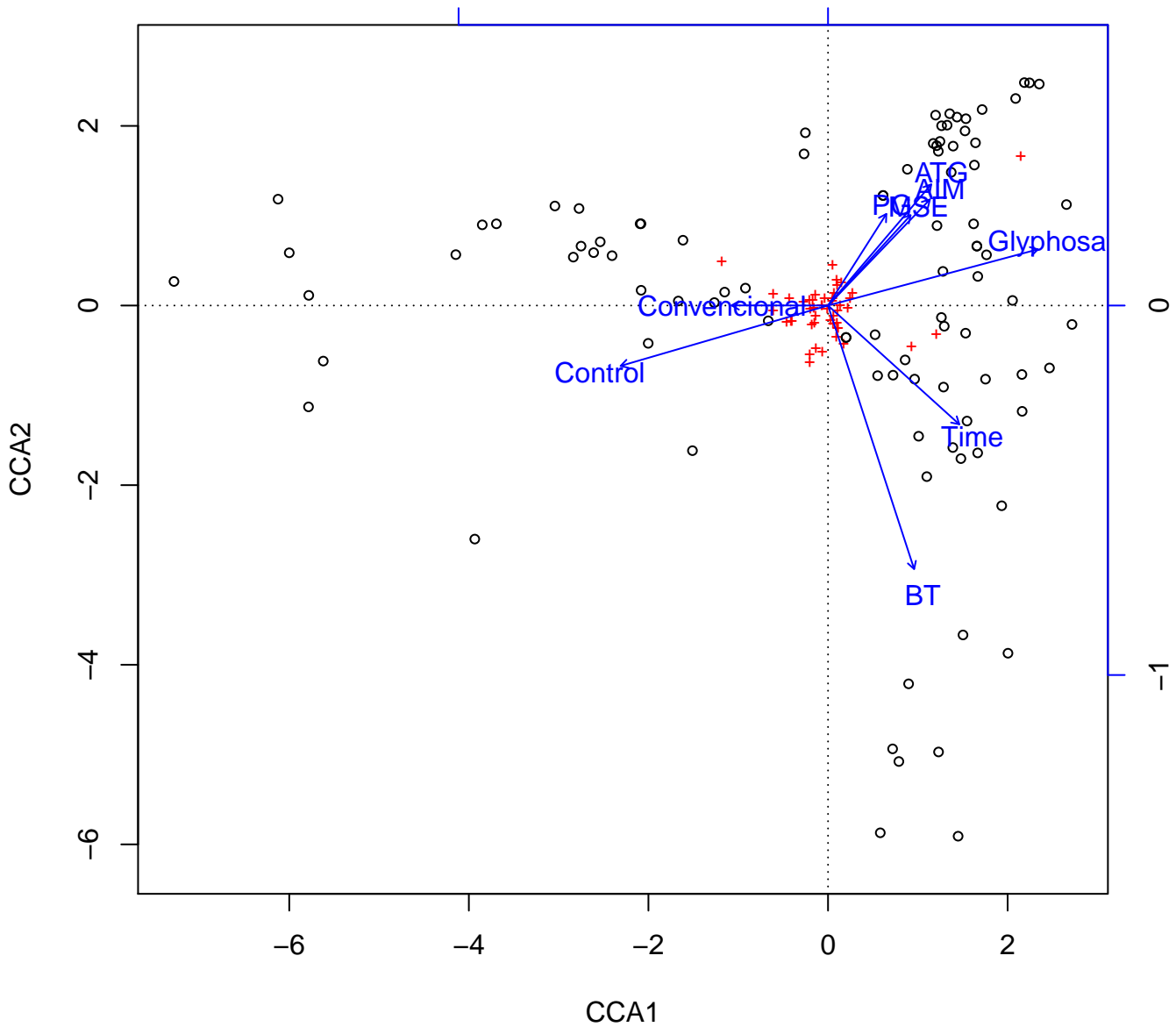

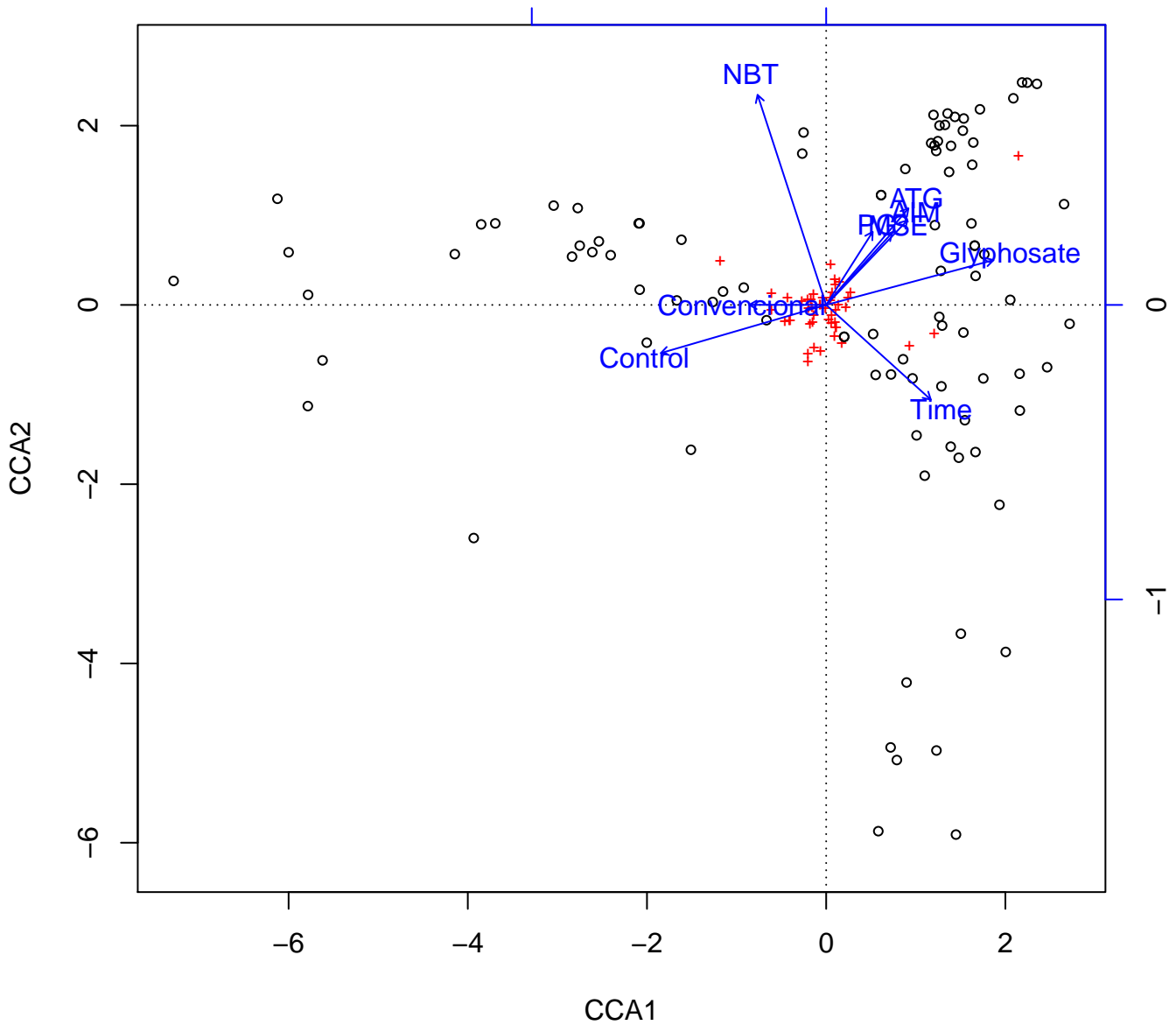

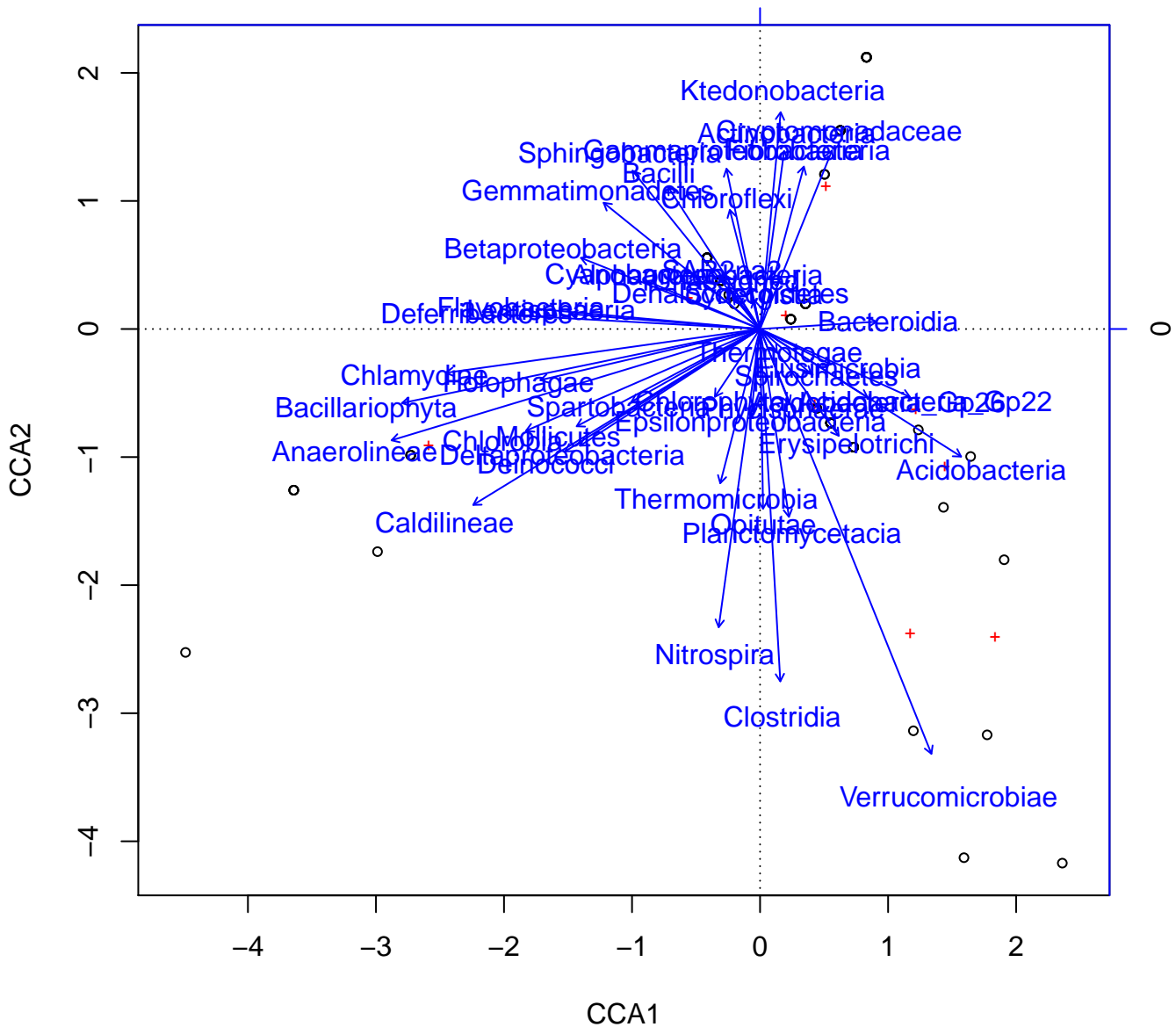

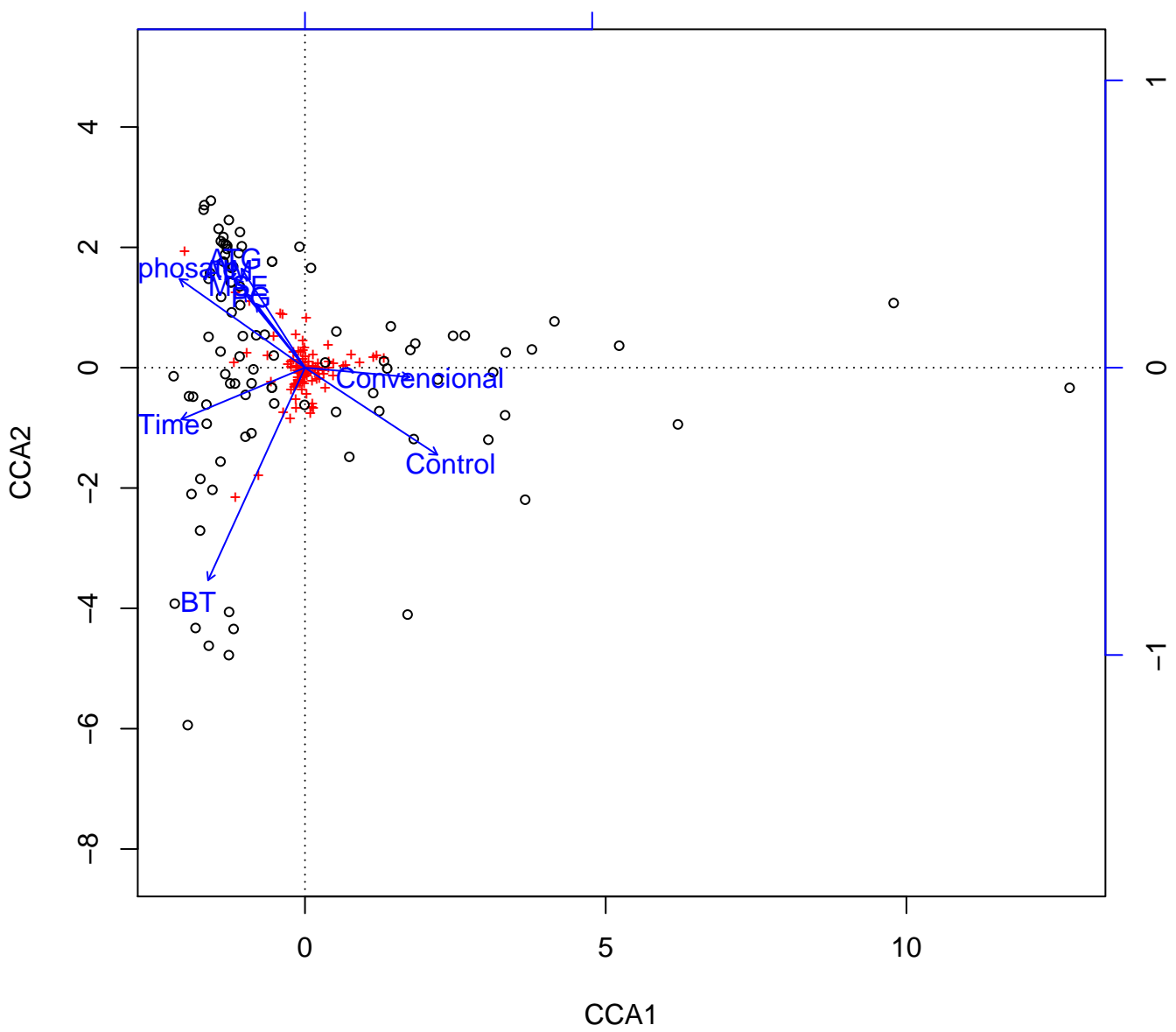

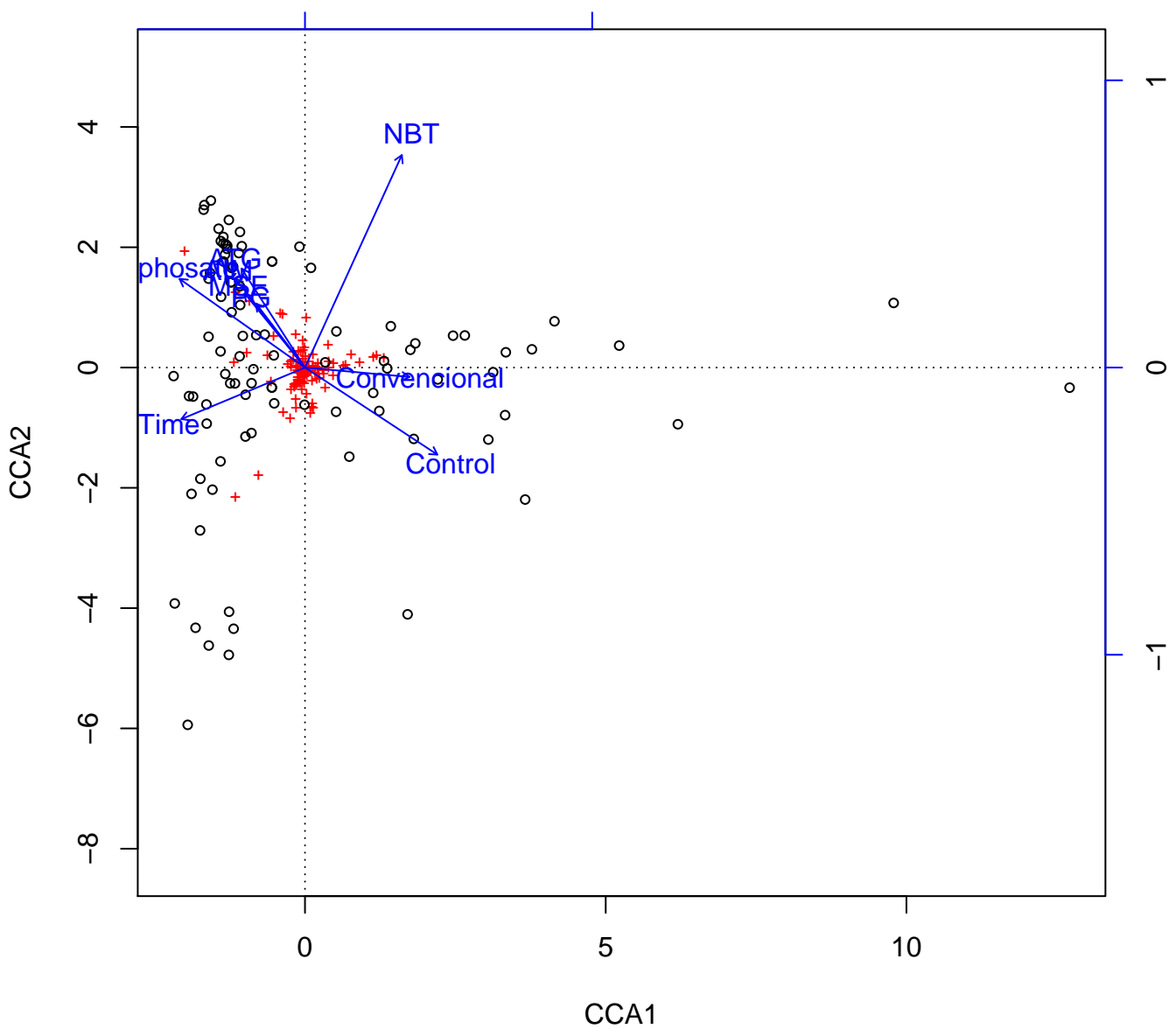

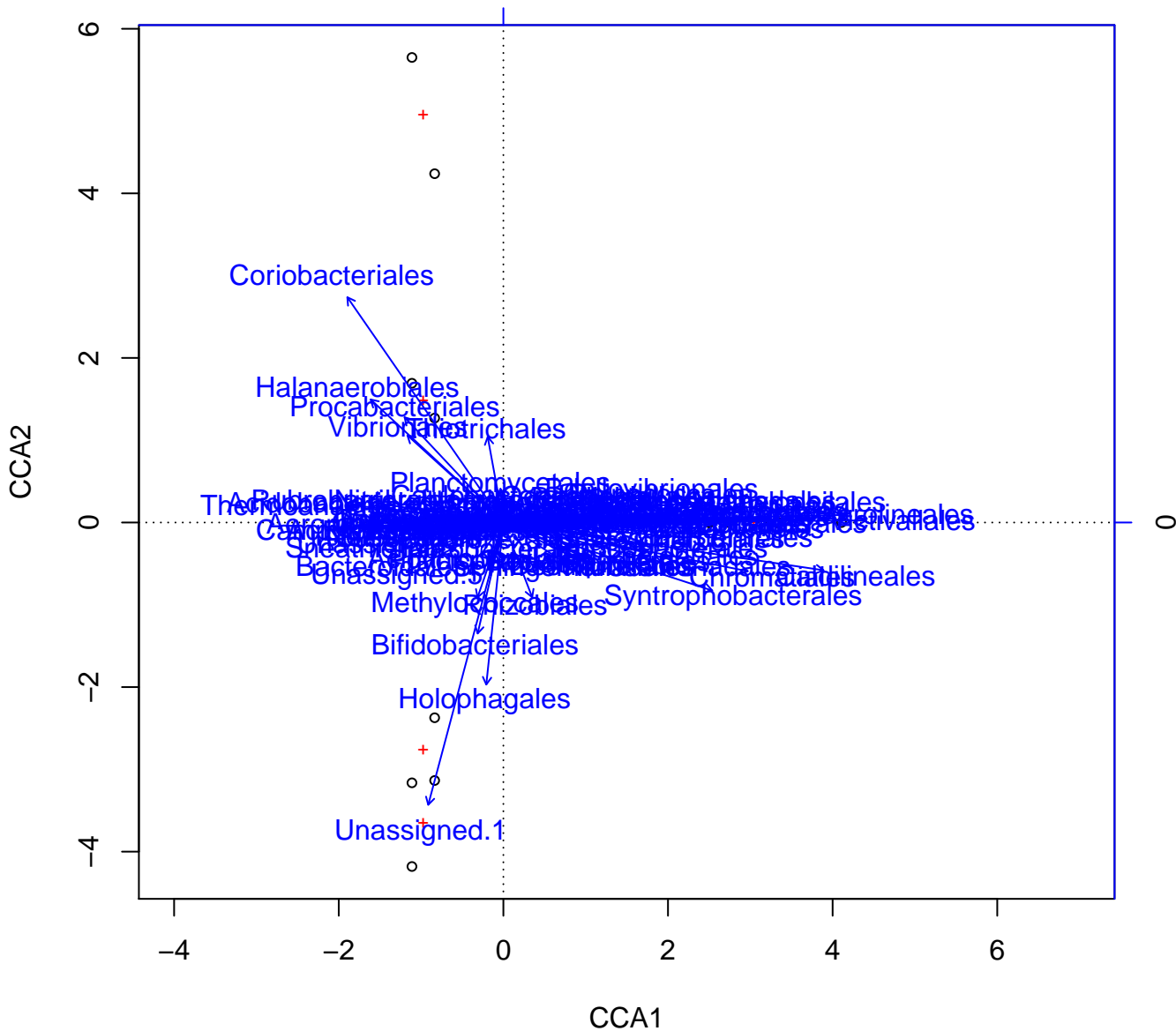

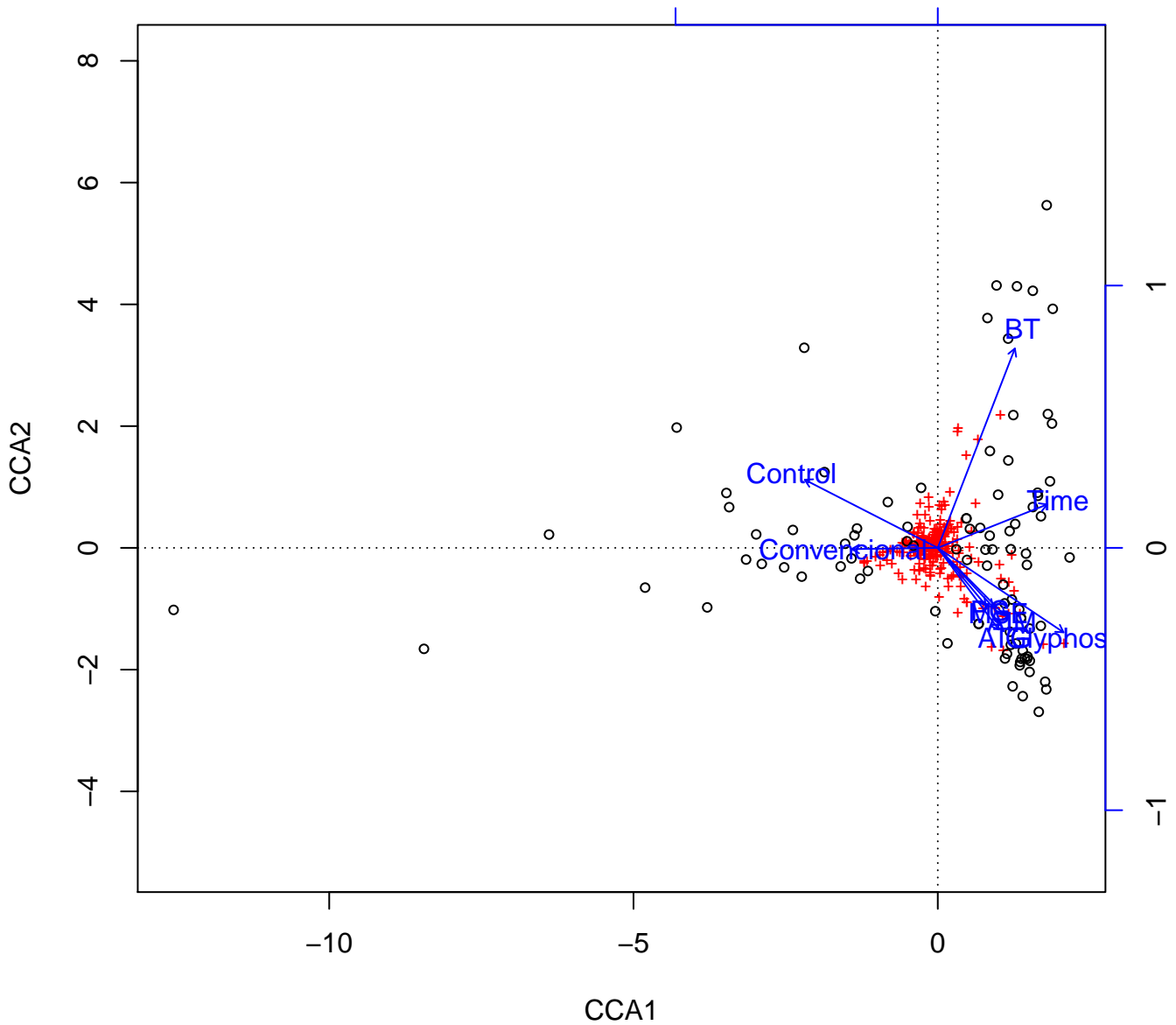

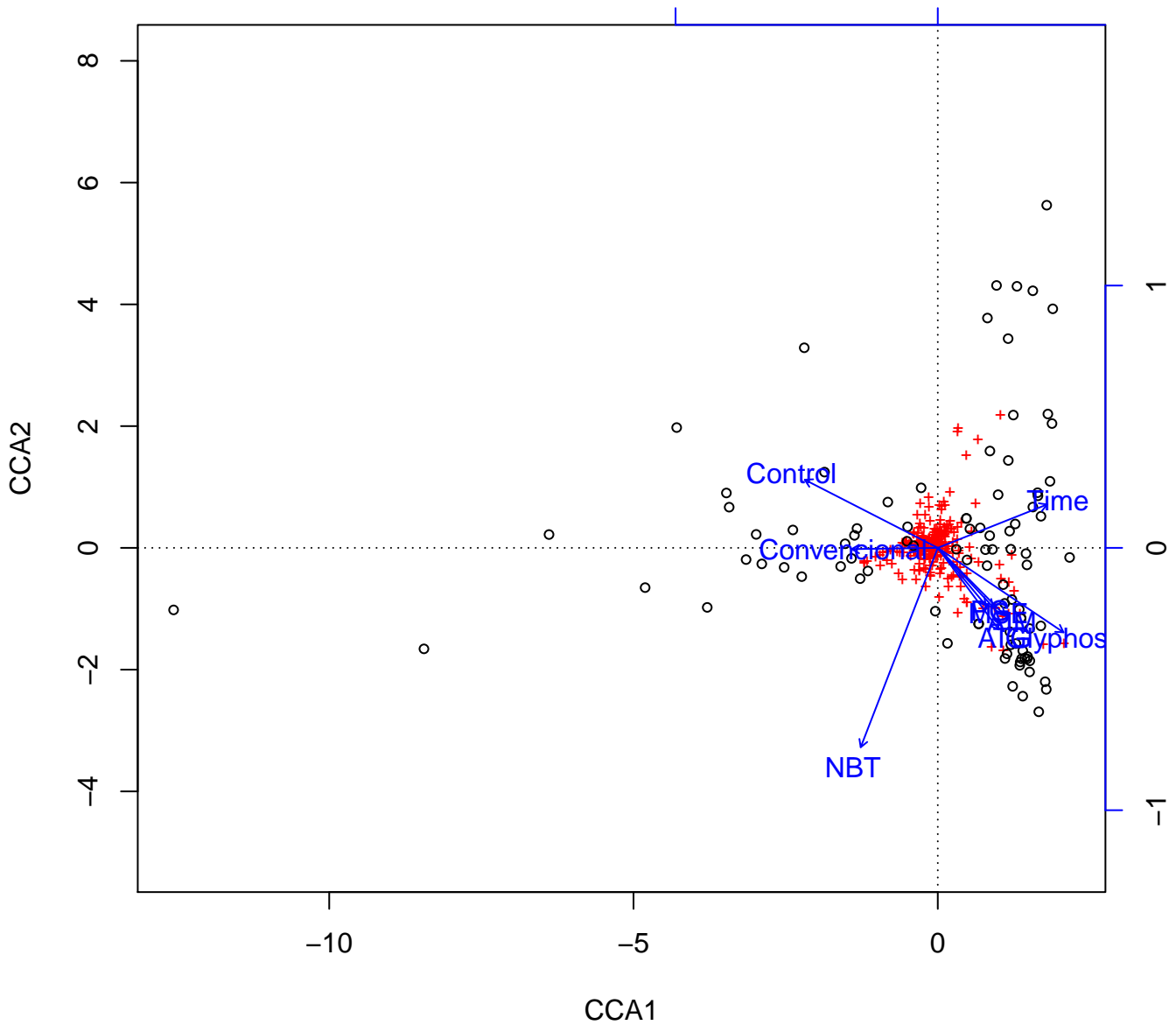

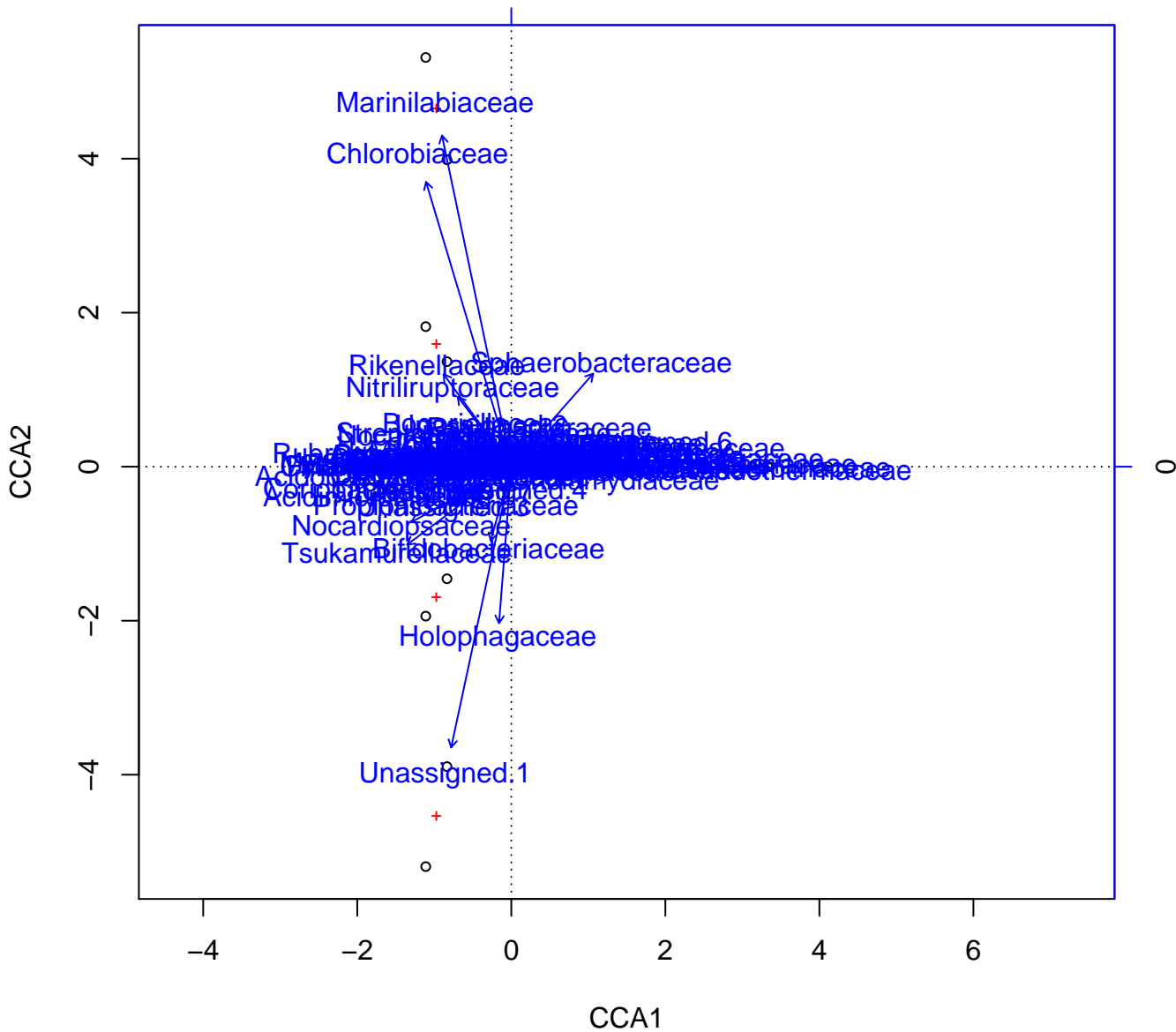

Supplement: S1 Appendix — The results of CCA and Indicator Value analyses obtained with each of the three classification methods are provided in separate subfolders as a compressed Zip archive. (ZIP) [file pone.0165204.s001.zip › S2_folder_R_analyses/rtax/cca/rtax_CCA_R_plots.pdf]
